# Phylogeography of *Ostreopsis* along West Pacific Coast, with Special Reference to a Novel Clade from Japan

**DOI:** 10.1371/journal.pone.0027983

**Published:** 2011-12-02

**Authors:** Shinya Sato, Tomohiro Nishimura, Keita Uehara, Hiroshi Sakanari, Wittaya Tawong, Naohito Hariganeya, Kirsty Smith, Lesley Rhodes, Takeshi Yasumoto, Yosuke Taira, Shoichiro Suda, Haruo Yamaguchi, Masao Adachi

**Affiliations:** 1 Royal Botanic Garden Edinburgh, Edinburgh, United Kingdom; 2 Institute of Molecular Plant Sciences, School of Biological Sciences, University of Edinburgh, Edinburgh, United Kingdom; 3 Kochi University, Kochi, Japan; 4 The United Graduate School of Agricultural Sciences, Ehime University, Ehime, Japan; 5 Cawthron Institute, Nelson, New Zealand; 6 Japan Food Research Laboratories, Tama Laboratory, Tokyo, Japan; 7 Okinawa Institute of Science and Technology Evolutionary Systems Biology Unit, Okinawa, Japan; 8 University of the Ryukyus, Okinawa, Japan; University of New South Wales, Australia

## Abstract

**Background:**

A dinoflagellate genus *Ostreopsis* is known as a potential producer of Palytoxin derivatives. Palytoxin is the most potent non-proteinaceous compound reported so far. There has been a growing number of reports on palytoxin-like poisonings in southern areas of Japan; however, the distribution of *Ostreopsis* has not been investigated so far. Morphological plasticity of *Ostreopsis* makes reliable microscopic identification difficult so the employment of molecular tools was desirable.

**Methods/Principal Finding:**

In total 223 clones were examined from samples mainly collected from southern areas of Japan. The D8–D10 region of the nuclear large subunit rDNA (D8–D10) was selected as a genetic marker and phylogenetic analyses were conducted. Although most of the clones were unable to be identified, there potentially 8 putative species established during this study. Among them, *Ostreopsis* sp. 1–5 did not belong to any known clade, and each of them formed its own clade. The dominant species was *Ostreopsis* sp. 1, which accounted for more than half of the clones and which was highly toxic and only distributed along the Japanese coast. Comparisons between the D8–D10 and the Internal Transcribed Spacer (ITS) region of the nuclear rDNA, which has widely been used for phylogenetic/phylogeographic studies in *Ostreopsis*, revealed that the D8–D10 was less variable than the ITS, making consistent and reliable phylogenetic reconstruction possible.

**Conclusions/Significance:**

This study unveiled a surprisingly diverse and widespread distribution of Japanese *Ostreopsis*. Further study will be required to better understand the phylogeography of the genus. Our results posed the urgent need for the development of the early detection/warning systems for *Ostreopsis*, particularly for the widely distributed and strongly toxic *Ostreopsis* sp. 1. The D8–D10 marker will be suitable for these purposes.

## Introduction

Some phytoplankton species are known to be a nuisance: they occasionally generate high biomass that can deplete dissolved oxygen and/or be lethal to filter feeders as their gills/filters are choked up with the mass of cells. Furthermore, among these phytoplankton, some species produce toxic substances that seriously threaten public health and cause enormous economic losses to fisheries as well as tourism. These harmful algal blooms (HAB) have been expanding globally in recent years and their frequency and extent have been increasing [1], [2]. One of the most commonly reported HAB organisms along coastal area are the dinoflagellates, a large protist group producing diverse toxic compounds, such as saxitoxin, dinophysistoxin, brevetoxin, ciguatoxin, maitotoxin and palytoxin. Most species of a dinoflagellate genus *Ostreopsis* are potential producers of palytoxin or its derivatives, palytoxin being the most potent non-proteinaceous compound reported so far [3]. Clupeotoxism, an often fatal illness to human, can be caused by shellfish and bottom-feeders (e.g. Serranidae fishes) that have sequestered these toxins [4], [5], [6], [7].

On the Italian coast around the city of Genoa, a total of 209 people who spent time on or near beaches between 17 and 26 July 2005 sought medical treatment for symptoms such as rhinorrhoea, cough, fever, bronchoconstriction with mild breathing difficulties, wheezing, and, in a few cases, conjunctivitis. Sea water samples from around beaches were found to have high densities of *Ostreopsis ovata*, up to several thousands cells/l of sea water and hundreds of thousands of cells/g on macroalgal samples [8]. The analysis of water, plankton and macroalgal samples demonstrated the presence of putative palytoxin [9]. In New Zealand *O. siamensis* blooms caused mass mortalities of sea urchins, *Evechinus chloroticus* [10], although no incidents of human illness have been associated with these blooms. Given the growing number of the reports on *Ostreopsis* blooms in the world ([10], [11] and refs therein) and the fact that the palytoxin-like poisoning is rapidly increasing along Japanese coastal area [5], [12], it is reasonable to assume that increases in the frequency and expansion of the scale of *Ostreopsis* blooms are also in progress along Japanese coast. Thus, there is an urgent need to reveal the distribution, diversity and toxicity of *Ostreopsis* in this area, and to develop the early detection/warning system for the genus.

Taxonomic studies of the genus *Ostreopsis* were undertaken based on its morphological features. By virtue of early workers, the knowledge of *Ostreopsis* morphology, particularly thecal fine structure under scanning electron microscopy (SEM), has greatly increased (e.g. [13], [14], [15], [16], [17], [18], [19], [20], [21], [22], [23]). However, unequivocal identification of *Ostreopsis* species under light microscopy (LM) is challenging since the distinguishing characteristics available are only the cell outline and these dimensions are overlapping across the species and have intraspecific variability [22], hampering the use of LM-based information, for instance, to monitor aquaculture areas. Therefore, genetic markers that can assuredly discriminate species, ideally down to strain level, will be essential to overcome the identification difficulties. Besides, genetic markers can also be a powerful tool for investigating the distribution and diversity of *Ostreopsis*, as well as for the subsequent establishment of a detection system for monitoring purposes. So far phylogeographic studies on *Ostreopsis* have only been carried out on Mediterranean, West Atlantic and south Pacific specimens [20], [24], [25], [26] and no material from Japanese and New Zealand coasts has been examined by means of genetic markers.

A phylogeographic study of *Ostreopsis* was firstly carried out by Pin et al. [24] which revealed, using ITS and 5.8 S region of rDNA, that Malaysian *O. ovata* comprised a Malacca Straits clade and a S. China Sea clade, having a high level of sequence divergence between them, while a low level of divergence within clades. They also found that *O. lenticularis* was genetically distinct from *O. ovata*. Subsequently Penna et al. [20] analyzed W. Mediterranean and S.W. Atlantic (Med/Atl) *Ostreopsis* using the ITS. An additional clade of *O. ovata* was detected into which all their sequences were included with little sequence divergence among them, being a sister to Malaysian clones obtained by Pin et al. [24]. In their phylogenetic tree *O. ovata* was sister to *O.* cf. *siamensis* [20]. Recently Penna et al. [25] analyzed the genus again based on an extended sampling and reported a slightly altered ITS topology of *O. ovata* in that Med/Atl clade was grouped together with Malaysian S. China Sea clade, as a whole which was sister to a clade comprising Malaysian Malacca Straits/Indonesian Celebes Sea clades (S China/Mal/Ind). (In Penna et al.'s 2010 paper the clade was called *O.* cf. *ovata* due to its taxonomic uncertainty. Hereafter, we follow this provisional name.) On the other hand, the topology of *O.* cf. *ovata* clade based on D1–D2 region of large subunit (LSU) rDNA (D1–D2) was similar to that of the ITS tree by Penna et al. [20]. In their ITS tree a clade of *O. labens/lenticularis* diverged at first, followed by the separation into *O.* cf. *ovata* and *O. siamensis* [25].

In the present study the phylogeography of *Ostreopsis* was examined based on the materials mainly collected from New Zealand and also from the southern part of Japan where palytoxin-like poisonings have increasingly been reported in recent years. The toxicity of some representative clones was tested by mouse bioassay. We obtained sequences of D8–D10 region of LSU rDNA (D8–D10). This region is informative enough to resolve intra-specific relationships, but it is moderately conserved so that the alignment is straightforward. Moreover, the LSU region can readily be sequenced directly, allowing relatively large-scale screening (>200 in this study) of specimens. Additionally we also sequenced the ITS for selected clones to compare the phylogenetic positions of our clones with sequences obtained by previous studies [20]; [24]; [25]. We compared the results of the phylogenetic analyses and discussed their suitability for the further use.

## Results

In the present study we collected 65 samples (Table S1) from which 216 clonal cultures of *Ostreopsis* were successfully established, and additional 7 clones were purchased/provided (Table S2). Although we made an attempt to identify our clones primarily based on LM, confident identification was difficult as taxonomic characteristics used at the rank of species, i.e. thecae arrangement and cell diameter, mostly overlapped across the species. In addition, their morphological plasticity under culture conditions hampered description of the original (i.e. unaffected by cultivation) shape. Thus, among 8 putative species recovered in phylogenetic analyses, 2 species identified with uncertainty were indicated as *O.* cf. *ovata* and *O.* cf. *siamensis*, and the others were left unidentified as *Ostreopsis* sp. 1–6, assuming that each clade separated with long branches recovered in the phylogenetic analyses represented independent species.

### D8–D10 analyses

The D8–D10 region was obtained from 222 clones of *Ostreopsis* and 1 clone of *Coolia* sp. for use as an outgroup. All the D8–D10 phylogenetic analyses, using 3 alignment algorithms, i.e. MAFFT, Muscle and ClustalW, and 2 optimal criteria, i.e. Maximum likelihood (ML) and Bayesian inference (BI), resulted in substantially the same topology (Fig. S1) recovering 4 major clades of Japanese clones; *O.* cf. *ovata* (clade A), *Ostreopsis* sp. 1 (clade B), *Ostreopsis* sp. 5 (clade C) and *Ostreopsis* sp. 6 (clade D). The only difference among the analyses were the position of the root. In all the BI analyses and the ML with ClustalW alignment (ML-ClustalW), the root was placed between the clade comprising *Ostreopsis* sp. 5 and *Ostreopsis* sp. 6 and the others, while the tree was rooted between *Ostreopsis* sp. 3 (CAWD184) and 4 (CAWD179) with ML-MAFFT or between *Ostreopsis* sp. 3 and the others with ML-Muscle. Unless noted otherwise hereafter we discuss the D8–D10 phylogeny based on the ML-ClustalW, and nodal supports on the BI were taken from the BI-ClustalW as there were little difference in these values among BI analyses.

**Figure 1:**
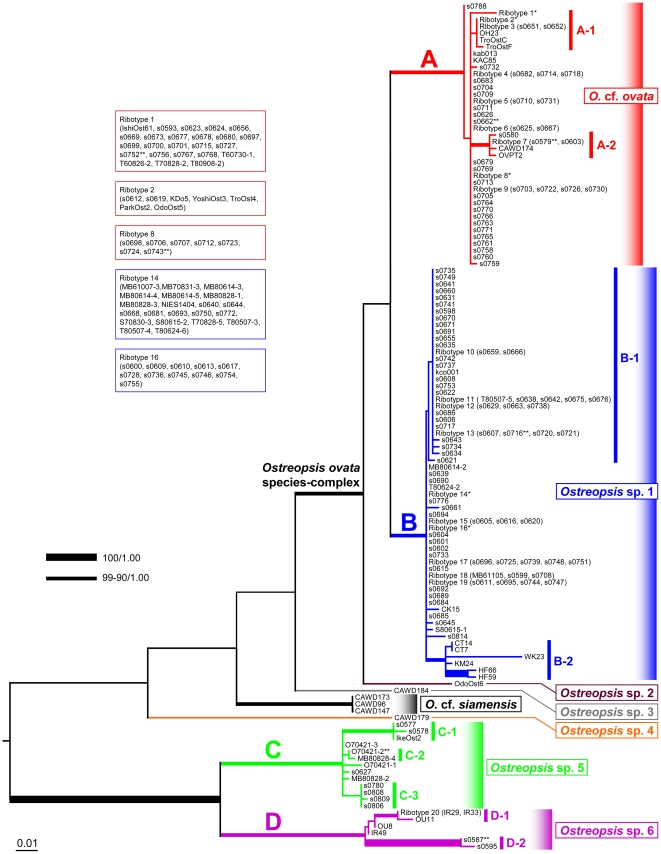
Maximum likelihood phylogeny of *Ostreopsis* inferred from D8/D10 sequence aligned with ClustalW. Tree is rooted with *Coolia* as outgroup but pruned for simplify. See Fig. S1 for original topology. Major clades found in Japanese coast are particularly noted as clade A–D, and their subclades are also indicated (e.g. A-1, A-2). Nodes with strong supports (bt/pp) are shown as thick lines. Sequences shared with more than one clone has been removed for phylogenetic analysis leaving 1 sequence as a representative of a ribotype. *Ribotype containing many clones are separately displayed at left hand. **Clone (currently being) used for various experiments.

In the ML-ClustalW (Fig. 1), the *Ostreopsis* clade was firstly bifurcated into 2 subclades. The first smaller one, bearing the highest nodal supports, i.e. bootstrap (bt) value of 100 and Bayesian posterior probability (pp) of 1.00, was further divided into *Ostreopsis* sp. 5 and *Ostreopsis* sp. 6. The monophyly of *Ostreopsis* sp. 5 clade was statistically supported (bt: 90, pp: 1.00). All the clones in this clade had a unique sequence. *Ostreopsis* sp. 5 clade comprised 13 clones, containing 1 well-defined clade C-1 (bt: 99, pp: 1.00, for IkeOst2, s0577 and s0578) and 2 weak clades C-2 (bt: 48, pp: 0.72, for MB80828-4 and O70421-2) and C-3 (bt: 22, pp: 0.52, for s0780, s0806, s0808 and s0809). *Ostreopsis* sp. 6 (bt: 90, pp: 1.00) was consisted of 7 clones that were subdivided into 2 robust clades: one with 5 clones involving small clade D-1 (bt: 98, pp: 1.00, for IR29, IR33 and OU11) and OU8 and IR49, another was a clade D-2 (bt: 100, pp: 1.00, for s0587 and s0595) which was highly diverged separated by a long branch.

Among the larger subclade being sister to the smaller one for *Ostreopsis* sp. 5 and *Ostreopsis* sp. 6, clones collected from Oceania area diverged ladder-like fashion such as *Ostreopsis* sp. 4 (CAWD179 from Australia), followed by an *O.* cf. *siamensis* clade (CAWD 96, CAWD147 and CAWD173 from New Zealand), and then *Ostreopsis* sp. 3 (CAWD184 from Cook Isls.).

The rest of clones formed a clade of *O. ovata* species-complex (bt: 90, pp: 1.00) in that all the clone shared similar morphology that could be identified as *O. ovata*. After the first divergence of *Ostreopsis* sp. 2 (OdoOst6), the clones were divided into *Ostreopsis* cf. *ovata* (bt: 97, pp; 1.00) and *Ostreopsis* sp. 1 (bt: 95, pp; 1.00).

A clone s0788 branched off at the base of *Ostreopsis* cf. *ovata*. The relationships for the rest of the clones were largely unresolved, except for a moderately supported clade A-1 (bt: 67, pp: 0.90) for the clones collected from subtropical (s0651, s0652, KDo5, YoshiOst3, TroOst4, ParkOst2, OdoOst5, OH23, TroOstC, OroOstF) and temperate (s0612, s0619) areas A highly supported clade A-2 (bt: 95, pp: 0.99) comprised clones with geographically heterogeneous origin including Malaysia (OVPT2), Cook Islands (CAWD174) and Japan (s0579, s0580, s0603). Although *Ostreopsis* cf. *ovata* comprised 83 clones, many sequences were common as shared ribotypes so the numbers of the unique sequences in the clade was 39. Ribotype 1 was the most commonly found, shared by 24 clones dispersed widely in the southern part of Japan.

All the members in *Ostreopsis* sp. 1 were only collected from Japanese coastal areas. Among 121 clones in the clade, 2 subclades, B-1 (bt: 27, pp: -) and B-2 (bt: 96, pp: 1.00) were formed leaving the rest of 67 clones as a basal polytomy. The clade B-1 consisted of 39 clones with no internal phylogenetic structure, received no statistical support and was not recovered in all the BI analyses, although the ML-MAFFT and the ML-Muscle supported its monophyly (not shown). The clade B-2 was based on 6 clones collected from the middle to the northern part of Japan (Chiba: CT7, CT14, Wakayama: WK23, Kyoto: KM24, Hokkaido: HF59, HF66) exhibiting high degree of sequence variability. *Ostreopsis* sp. 1 comprised 62 unique ribotypes, in that ribotype 14 was predominant, shared by 21 clones that were mostly collected from Shikoku, except for 2 clones, NIES1404 (Hachijojima) and s0693 (Kyushu).

### ITS analyses

ITS sequences were obtained from 25 clones of *Ostreopsis* and analyzed together with 76 public sequences retrieved from GenBank. The highly variable nature of the ITS made the *Ostreopsis* sequences impossible to be aligned with *Coolia*, which had routinely been used as an outgroup for the previous phylogenetic analyses on *Ostreopsis* (e.g. [20]). Furthermore, even within the genus *Ostreopsis* the sequence variability was so high that the alignments produced with different algorithms (viz., MAFFT, Muscle and ClustalW) resulted in different topologies (Fig. S2), although little difference was detected between the different optimally criteria, ML and BI.

In all the ITS analyses 3 clades for *Ostreopsis* sp. 1, *Ostreopsis* sp. 5 and *Ostreopsis* sp. 6 recovered in the D8–D10 analyses were constantly found as monophylies. On the other hand, *O.* cf. *ovata* formed a robust clade in ML and BI trees reconstructed with dataset aligned by MAFFT, but was paraphyletic with the Muscle and the ClustalW datasets. The phylogenetic positions of *Ostreopsis* sp. 3 (CAWD184) and *Ostreopsis* sp. 4 (CAWD179) with respect to the *O.* cf. *siamensis* clade were variable depending on the alignment algorisms (Fig. S2).

**Figure 2:**
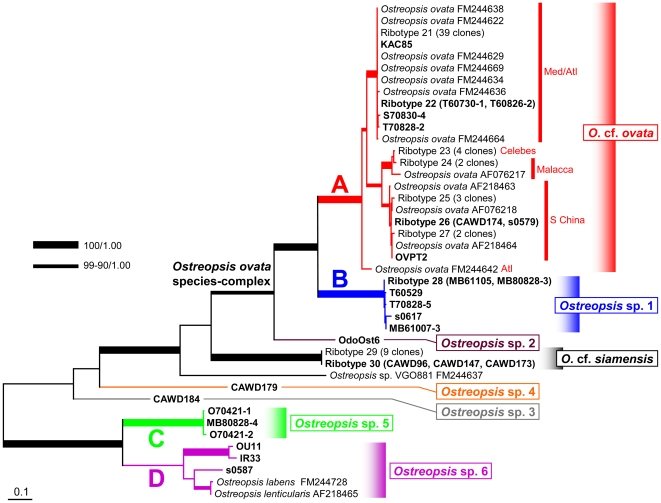
Maximum likelihood phylogeny of *Ostreopsis* inferred from ITS sequence aligned with MAFFT. Major clades found in Japanese coast are particularly noted as clade A–D. Geographic origins of *O.* cf. *ovata* clone are indicated. Sequences of bold clone are obtained in this study. Note tree is unrooted but displayed as rooted fashion. See caption in Fig. 1 for detail and Fig. S2 for original topology.

The tree reconstructed from the MAFFT alignment was virtually the same with the topology recovered in all the D8–D10 phylogenies. In Fig. 2 we present the result of the ITS phylogenetic analysis based on ML-MAFFT, supposing the root position is the same with the D8–D10 tree, namely between a smaller subclade for *Ostreopsis* sp. 5 and 6 and a larger clade for the others.

Given the higher substitution rate of the ITS region, the resolution of the intra-clade relationship was greater than that of the D8–D10, most prominently within *O.* cf. *ovata* clade. Unlike the D8–D10 tree, the members of *O.* cf. *ovata* were clearly divided into 3 clades: one sequence (FM244642, clone VGO614 collected from Madeira, East Atlantic [25]) branched off firstly and then divided into S China/Mal/Ind clade (bt: 98, pp: 1.00) and Med/Atl clade (bt: 100, pp: 1.00) (see introduction for the clade names), the former included one Japanese clone (s0579) and the latter also included 4 Japanese clones (T60730-1, T60826-2, T70828-2 and S70830-4) collected in this study.

Our 3 clones collected from Japanese subtropical (OU11, IR33 and s0587) formed a clade along with 2 GenBank sequences annotated as *O. lenticularis* (AF218465) and *O.* cf. *labens* (FM244728): however, in this study we left them unidentified as *Ostreopsis* sp. 6 since morphologically we were unable to identify them. Between AF218465 and FM244728 there were 3 substitutions in 377 bp, thus, uncorrected genetic distance (*p*) was 0.0079. The positions and nucleotides of the variable sites in the alignment (AF218465∶FM244728) were 261 (G∶T), 269 (A∶T), 283 (G∶T), all within the ITS2 region. The retrieved sequences were the same length and could not be aligned without introducing gaps. Because 3 sites were (G or A∶ T), it was clear that no complementally base changes (CBCs) or hemi-CBCs occurred in the ITS2. The ITS sequences of OU11 and IR33 (corresponded to clade D-1 in D8–D10 tree) and s0587 (clade D-2) were extracted from the full dataset and re-aligned with MAFFT to obtain the *p* value of the ITS between the clade D-1 and D-2. This alignment consisted of only 3 sequences without any distantly related sequence, rendering more reliable estimation of the number of substitutions possible. As a result there are 74.5 substitutions in 486 bp and the *p* = 0.222.

### Sequence analyses

D8–D10 and ITS alignments produced by 3 different alignment algorisms are compared in Table 1. The relatively conserved sequence of the D8–D10 rendered the alignment straightforward, yielding almost the same alignment length, number of informative sites, and less variable parameters for the evolutionary model.

**Table 1:** Details of the datasets for phylogenetic analyses.

		No of informative sites		Parameters for GTR model*		Substitution rates**					Bayesian inference	
	Length (bp)	Parsimony	Phylogenetically	G	I	A–C	A–G	A–T	C–G	C–T	Generations	Burn-in trees***
*D8–D10*												
MAFFT	838	121	81	0.6845	0.5013	1.3142	2.4681	1.0181	0.4596	4.9010	5,000,000	31,900
Muscle	839	119	76	0.7010	0.5397	1.3313	2.4808	1.1668	0.4857	4.6891	5,000,000	38,900
ClustalW	840	122	81	0.7950	0.5395	1.2852	2.4354	1.1355	0.6269	4.4491	4,000,000	22,900
*ITS*												
MAFFT	560	286	81	1.2488	0.1236	0.7764	1.7469	0.6385	0.4378	2.0153	2,000,000	4,000
Muscle	430	300	55	1.5864	0.1428	0.9229	1.9761	1.5545	0.3548	1.6342	3,000,000	13,000
ClustalW	466	284	50	0.7093	0	1.0341	2.4608	0.8419	0.3820	2.6934	2,000,000	5,000

*Both hLRTs and AIC favored GTR model for all the dataset.

**Calculated as G–T = 1.0000.

***Trees discarded as burn-in before ASDS below 0.01.

**Figure 3:**
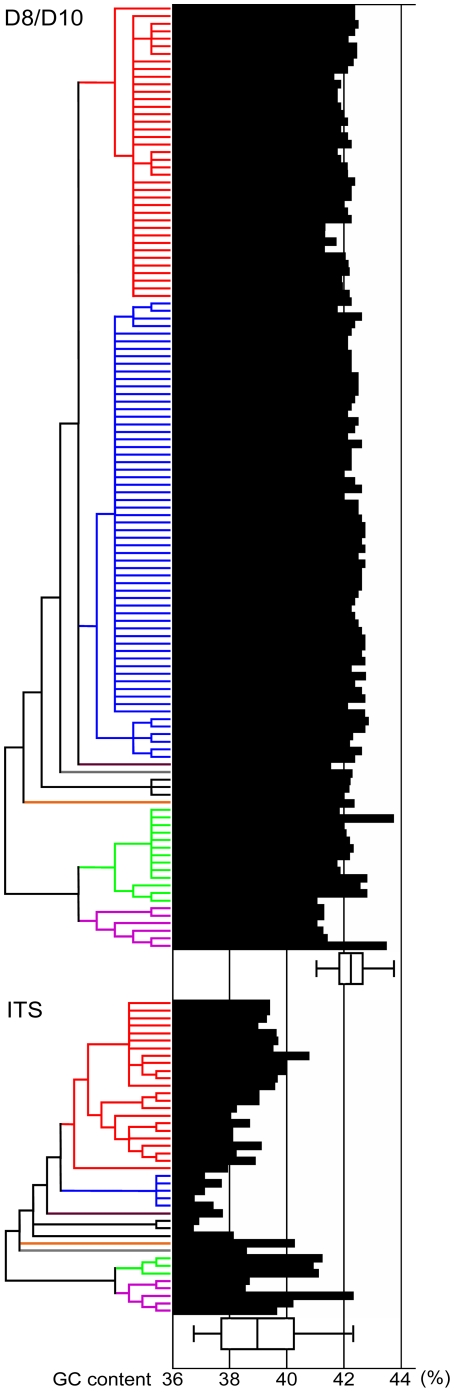
Comparison of genetic heterogeneity in *Ostreopsis* between D8/D10 and ITS. GC content of each sequence is shown with ML topologies taken from Figs. 1 and 2, which are condensed with cut-off value 50% by MEGA4. Box plot indicates minimum and maximum (horizontal bar), average (median vertical bar in box) and standard deviation above and below mean of the data (box width).

The sequence heterogeneity of the D8–D10 and the ITS was also compared by means of GC content and its standard deviations (Fig. 3). The average and the standard deviation (SD) of the D8–D10 and the ITS are 42.25% (±0.41) and 38.98% (±1.29) respectively. The SD of the ITS sequence heterogeneity was more than three fold greater than that of the D8–D10, indicating high heterogeneity in the ITS. *Ostreopsis* sp. 1 (blue clade in Fig. 3) showed remarkably lower GC content in the ITS while D8–D10 displayed more or less constant values across all lineages.

Molecular diversity was assessed by the net number of nucleotide differences, based on the D8–D10 and the ITS (Table 2). Both analyses suggested that each clade was highly differentiated, with the greatest divergence between OdoOst6 (a clone diverged at the root of the *O. ovata* species-complex clade) and *Ostreopsis* sp. 6. Clades between *O.* cf. *ovata* and *Ostreopsis* sp. 1 had the lowest divergent index. No inter-clade indices were lower than intra-clade indices, in which *O.* cf. *siamensis* was the lowest followed by *Ostreopsis* sp. 1.

**Table 2:** Estimates of evolutionary divergence over D8–D10 (top, ClustalW alignment) and ITS (bottom, MAFFT alignment) among and within clades of *Ostreopsis*.

	*O.*cf. *ovata*	*O.* sp. 1	*O.* sp. 2	*O.* sp. 3	*O.* cf. *siamensis*	*O.* sp. 4	*O.* sp. 5	*O.* sp. 6
*O.* cf. *ovata* (Clade A)	1.11±0.41							
	21.94±2.72							
*Ostreopsis* sp. 1 (Clade B)	17.58±3.76	1.01±0.22						
	62.23±6.38	5.00±1.35						
*Ostreopsis* sp. 2 (OdoOst6)	25.00±4.76	21.46±4.56	NA					
	100.35±7.51	115.80±8.52	NA					
*Ostreopsis* sp. 3 (CAWD184)	30.67±4.96	26.41±4.80	32.00±5.24	NA				
	101.57±8.21	105.80±8.29	137.00±9.28	NA				
*O.* cf. *siamensis*	33.92±5.53	34.51±5.60	38.00±6.11	32.00±5.17	<0.01			
	104.54±7.86	112.20±8.43	127.50±9.06	136.00±9.44	<0.01			
*Ostreopsis* sp. 4 (CAWD179)	58.23±7.16	60.34±7.17	61.00±7.38	52.00±6.62	55.00±7.09	NA		
	120.13±8.43	113.80±8.40	145.00±9.35	108.00±8.49	144.00±9.36	NA		
*Ostreopsis* sp. 5 (Clade C)	74.36±7.51	74.86±7.59	77.15±7.97	69.08±7.20	70.56±7.35	71.92±7.30	4.54±1.10	
	111.36±8.32	128.73±8.72	149.33±9.30	110.33±8.68	137.67±9.10	115.33±8.58	2.67±1.23	
*Ostreopsis* sp. 6 (Clade D)	73.04±7.54	79.36±8.10	79.83±8.03	73.83±7.88	75.17±7.78	77.83±7.96	41.00±5.92	9.40±1.93
	128.88±7.24	130.92±7.58	158.20±7.79	140.20±7.92	138.40±7.93	149.20±8.24	91.53±7.22	51.00±4.66

Data are mean pairwise nucleotide differences within species (on the diagonal), and net pairwise differences between species (below diagonal). Std. Err. calculated on 1,000 bootstrap replicates. NA, not applicable.

### Distribution

**Figure 4:**
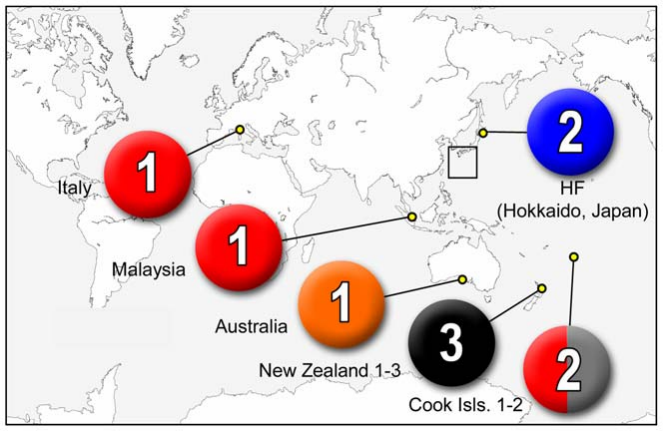
Geographic distributions of *Ostreopsis* plotted on world map. Pie chart illustrates species composition of each sample, in that total number of clones is indicated. Sample name is given below pie chart. Each color corresponds to a clade in phylogenetic trees in Figs. 1 and 2, i.e. red: O. cf. *ovata*, blue: *Ostreopsis* sp. 1, brown: *Ostreopsis* sp. 2, black: O. cf. *siamensis*, orange: *Ostreopsis* sp. 4, green: *Ostreopsis* sp. 5, purple: *Ostreopsis* sp. 6.

**Figure 5:**
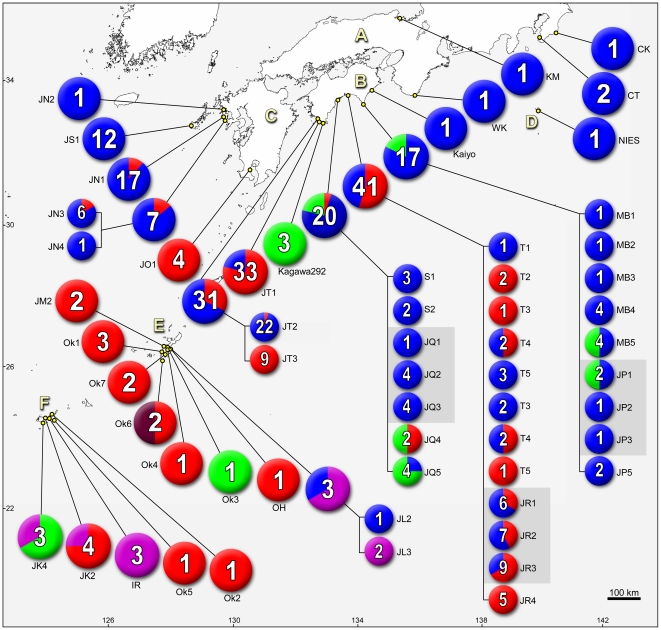
Geographic distributions of *Ostreopsis* plotted on map showing middle-southern part of Japan. Enlargement of a part enclosed by an open square in Fig. 4. See caption in Fig. 4 for more detail. For series of samples collected multiple times at a same site, breakdown of each sample is shown in small pies that are summarized in large one. Each island is marked as A: Honshu (main isl.), B: Shikoku, C: Kyushu, D: Hachijojima, E: Okinawa (main isl.), F: Ishigaki, Kohama and Iriomote (from right to left).

The occurrence and the species composition plotted onto the world map illustrated that our *Ostreopsis* clones collected from outside Japan contained no *Ostreopsis* sp. 1, *Ostreopsis* sp. 2, *Ostreopsis* sp. 5 or *Ostreopsis* sp. 6 (Fig. 4). An enlarged view of the mid-southern part of Japan in Fig. 5, where islands A–D are in temperate and E–F in subtropical climates, showed that the distribution of *Ostreopsis* sp. 6 was strictly restricted to subtropical areas in Japan. Additionally, some tendencies were seen in the distribution of Japanese *Ostreopsis* such as; *O.* cf. *ovata* was mainly distributed through the subtropical region, and the southern part of the temperate region of Japan, whereas *Ostreopsis* sp. 1 was more abundant in temperate areas. The distribution of *O.* cf. *ovata* and *Ostreopsis* sp. 1 largely overlapped in Japan.

The diversity of *Ostreopsis* in Okinawa Island was surprisingly high, yielding 5 species, *O.* cf. *ovata*, *Ostreopsis* sp. 1, *Ostreopsis* sp. 2, *Ostreopsis* sp. 5 and *Ostreopsis* sp. 6 from a single island (E in Fig. 5).

Species composition varied from sample to sample even within a series of samples collected from the same site (see circles showing breakdown with smaller circles in Fig. 5. Note: here we define the term *sample* for a subset of cells collected from a single seaweed substratum or a toe of plankton net). We also collected samples at slightly different positions, one to several meter distance of one another, but at the same site and date (enclosed by gray square in Fig. 5, JP1-3: Muroto, JR1-3: Tei, JQ1-3; Susaki). The results varied as we recorded different species composition at Muroto; the same composition but different proportion at Tei; fully consistent at Susaki. Substrata seaweeds were the same in Muroto and Tei, while different to one another in Susaki (Table S1).

**Figure 6:**
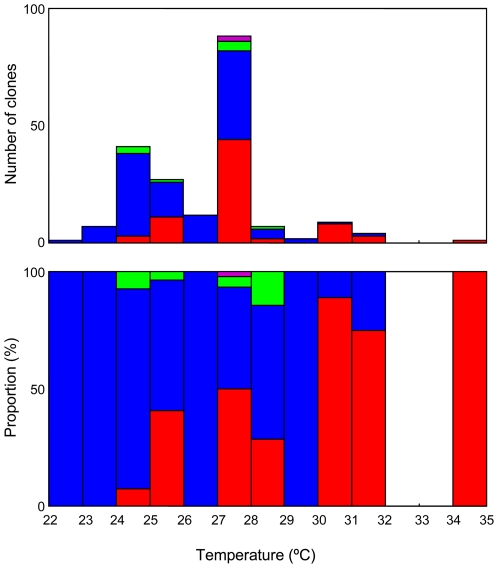
Comparison of *Ostreopsis* occurrence and water temperature. Absolute number (top) and relative abundance (bottom) of cells. Each color corresponds to a clade in phylogenetic trees in Figs. 1 and 2, i.e. red: O. cf. *ovata*, blue: *Ostreopsis* sp. 1, brown: *Ostreopsis* sp. 2, black: O. cf. *siamensis*, orange: *Ostreopsis* sp. 4, green: *Ostreopsis* sp. 5, purple: *Ostreopsis* sp. 6.

We compared the occurrence of each species with water temperature measured when the samples were taken. A slight tendency of the relative occurrence related to the temperature was found, i.e. *O.* cf. *ovata* was more abundant in warmer water, whereas *Ostreopsis* sp. 1 more commonly occurred in cooler water (Fig. 6).

### Mouse bioassay

Mice injected with the methanolic fractions of *Ostreopsis* clones displayed symptoms typical of palytoxin activity [7]. The highest toxicity level was monitored in *Ostreopsis* sp. 1 (clone s0716) in that three mice died from an extract of ×10^4^ cells within 24 h, while the other clones only showed toxicity with extracts of ×10^5^ cells, except for *Ostreopsis* sp. 5 (s0806) which had no mouse lethality (Table 3).

**Table 3:** Results of mouse bioassay (injection) for *Ostreopsis* clades.

Taxon (Clone)	Injected cell number	Mice death (after 48 h)
*O.* cf. *ovata* (s0715)	2.8×10^5^	−++^a^
	2.5×10^4^	−−+
*O.* cf. *ovata* (s0662)	3.5×10^5^	−++
	3.1×10^4^	−−+
*O.* cf. *ovata* (KAC85)	7.9×10^5^	+++
	7.1×10^4^	−−+
*Ostreopsis* sp. 1 (s0716)	5.0×10^5^	+++
	3.2×10^4^	+++
*Ostreopsis* sp. 5 (s0806)	2.0×10^5^	−−−
*Ostreopsis* sp. 6 (s0587)	3.6×10^5^	+++
	1.8×10^4^	−−+

a +++: 3/3 mice dead, −−−: no deaths.

### Morphology

**Figure 7:**
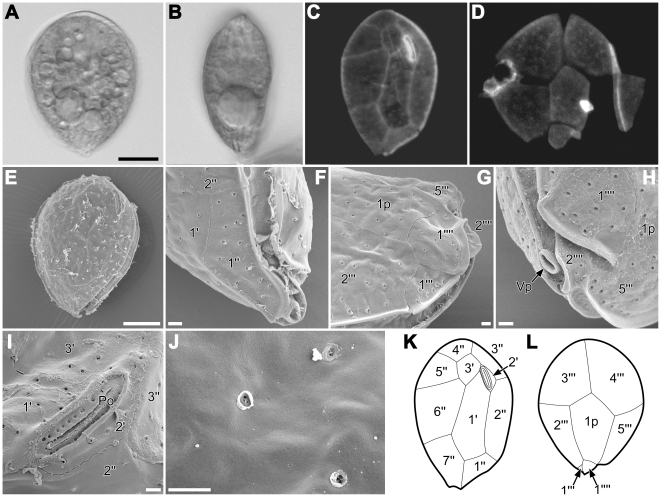
Morphology of *Ostreopsis* sp. 1 with LM (A, B), LM-epifluorescence (C, D), SEM (E–J) or line illustration (K, L). **A:** Living cell. **B:** Side view of living cell. **C:** Epithecal view. **D:** Hypothecal view. **E:** Epithecal view. **F:** Detail of ventral area from side view, showing ventral pore (Vp). **G:** Detail of ventral area from hypothecal view. **H:** Detail of ventral area from hypothecal view, showing Vp structure and associated 2″″ plate. **I:** Enlarged view of short, slightly curved pore plate (Po). **J:** Detail of cell surface, showing thecal pores. **K:** Epithecal view. **L:** Hypotheal view. Same magnification in A–D. Scale = 10 µm (A, E) or 1 µm (F–J).

Cell sizes were measured under LM on 3 clones representing each clade of *Ostreopsis ovata* species-complex, i.e. s0726 (*O.* cf. *ovata*), s0618 (*Ostreopsis* sp. 1) and OdoOst6 (*Ostreopsis* sp. 2). Most of the size ranges largely overlapped one another so that the clades could not be discriminated solely by the measurements (Table 4). Detailed observations with LM and SEM were undertaken on *Ostreopsis* sp. 1 (Fig. 7), the most dominant taxon in Japanese coastal waters during this study. Morphological characteristics of 3 clones fitted well into the species description of *O. ovata* as follows; live cells swam with a geotropic orientation, remaining attached to a bottom of the culture well; cells are anterio-posteriorly compressed and have golden plastid, and often have large vacuole(s) at the ventral end (Figs. 7A, B); the plate pattern was Po, 3′, 7″, 5′″, 2″″, 1p (Figs. 7C–L); thecal surface is smooth, ornamented with minute, evenly distributed pores (Figs. 7F–J). Occasionally we observed slightly distorted plates (not shown) and these are presumably deformities due to the extended cultivation. Morphologically we were unable to discriminate clones in the clade for *O. ovata* species-complex.

**Table 4:** Cell dimensions (µm) of *Ostreopsis.*

Clone (clone)	DV	W	AP
*O.* cf. *ovata* (s0726)	28.1±2.6 (n = 54)	21.2±2.8 (n = 54)	17.5±3.3 (n = 19)
*Ostreopsis* sp. 1 (s0618)	26.5±3.0 (n = 54)	19.4±2.6 (n = 50)	15.8±3.0 (n = 23)
*Ostreopsis* sp. 2 (OdoOst6)	29.1±4.4 (n = 52)	20.8±3.3 (n = 52)	21.2±2.4 (n = 22)

## Discussion

### *Ostreopsis* distribution

The genetic diversity of *Ostreopsis* revealed in this study was unexpectedly high since *Ostreopsis* spp. 1–5 were hitherto unreported. In particular, it was astonishing to uncover such a widespread distribution of *Ostreopsis* sp. 1 along the main research field, the middle to southern part of Japan. Furthermore, five species were found in this area (O. cf. ovata, *Ostreopsis* sp. 1, *Ostreopsis* sp. 2, *Ostreopsis* sp. *Ostreopsis* sp. 5 and *Ostreopsis* sp. 6), which is high compared with the other geographic regions (see review by Rhodes [11]). This diversity in Japanese waters might simply be explained by the intensive sampling in this study, viz. the more clones established, the more probability to encounter something new. It should, however, be noted that a “hot spot” of *Ostreopsis* diversity certainly exists: all the 5 species were recorded from the main island of Okinawa, a small island (1,206 km^2^) from where 15 clones were examined.

### Dispersal of *O.* cf. *ovata*

Our ITS tree based on MAFFT alignment exhibited the robust monophyly of *O.* cf. *ovata*, that is consistent with all the D8–D10 analyses, illustrating the initial divergence of a clone VGO614 (FM244642) with subsequent bifurcation into the Med/Atl clade and the Pac/Ind clade. The basal position of the Atlantic clone VGO614 made the S China/Mal/Ind clade nested within the Med/Atl clones, implying the possibility that a Med/Atl clone migrated into S China/Mal/Ind region and gave rise to the S China/Mal/Ind clade.

Noteworthy, 4 clones of Japanese *O.* cf. *ovata* (S70830-4, T60730-1, T60826-2, T70828-2) fell into the Med/Atl clade in the ITS tree, although we expected them to appear in the Pac/Ind clade taking their geographic origin into account. This topology strongly suggests that the geographic distance is not necessarily related to the genetic distances in *O.* cf. *ovata*, viz., Japanese *O.* cf. *ovata* might have migrated into Mediterranean and/or Atlantic oceans, or vice versa. The bush-like divergence and short branches in the Med/Atl clade indicates that the radiation of *O.* cf. *ovata* took place in a short period in recent times, compared with relatively resolved S China/Mal/Ind clade.

Penna et al. [25] explained the genetic homogeneity between Mediterranean and W. Atlantic populations with a gene flow maintained by the Canary and Equatorial Currents in an east to west direction, and by the Gulf Stream/Azores Currents and North Equatorial Counter Current from west to east. For Japanese and Med/Atl population, however, it is more reasonable to invoke anthropogenic pathways such as reported in *Alexandrium catenella* [27] or *Undaria pinnatifida* [28], given the lack of such direct link (i.e. oceanic current), despite most of the molecular data have led to a resurrection of transoceanic dispersal theories in recent years [29], [30]. The elucidation of the dispersal in *O.* cf. *ovata* will also shed light on the phylogeography of *O.* cf. *siamensis* which also exhibits a segregated distribution.

On the other hand, the connection with North Equatorial Current and/or Equatorial Counter Current might explain the distribution of S China/Mal/Ind clade: two of our clones, CAWD174 and s0579, collected from the Cook Islands and Iriomote Island, respectively, were embedded in a clade comprised of clones collected from Malaysian waters (PR01: AF218457, PR02: AF218459, PR04: AF218458, SA02: AF218460, SA04: AF218461, SA06: AF218463, PR03: AF076218, SA10: AF218464, obtained by [24], and OVPT2 by this study). A similar relationship can be seen in the D8–D10 tree, with lower resolution though, in which A-2 clade comprises of clones from Cook Islands, Malaysia and south Japan (collected from JN1, JK2 from JK4, see Fig. 5), the latter being connected with Kuroshio Current originating from North Equatorial Current. A finer molecular marker would be needed to further elucidate the phylogeographic history of *O.* cf. *ovata.*

### *Ostreopsis* sp. 1

This study unveiled the existence of *Ostreopsis* sp. 1 and its widespread distribution along Japanese coast. This unidentified species accounts for more than half of the total numbers of the clones examined during this study, i.e. 121 out of 223 clones. Contrary to globally dispersed *O.* cf. *ovata*, *Ostreopsis* sp. 1 had never been found in the other geographic region, despite particular attention has been paid to the occurrence of *Ostreopsis* in recent years [11].

This highly restricted distribution of *Ostreopsis* sp. 1 is rather curious – *O.* cf. *ovata*, *Ostreopsis* sp. 1 and *Ostreopsis* sp. 2 are morphologically indistinguishable and often sympatric, having almost the same opportunity for dispersal. One possibility is that their physiological features were affected by factors such as nutrient availability or response to different light intensity, as observed in marine cyanobacteria [31], [32] and the picoalga *Ostreococcus tauri* [33], or to susceptibility to different parasitic fungi as reported in diatoms [34]. Although we detected no sign of clade-specific growth characters in our culture, it is reasonable to assume the existence of such a physiological differentiation, as was discussed for a low temperate tolerant ecophenotype of *O. siamensis* by Pearce et al. [35]. Such an hypothesized effect would certainly make a marked difference to survival rates during the migration. As seen in Fig. 6 there is a slight tendency for *Ostreopsis* sp. 1 to be more abundant at lower temperatures compared to *O.* cf. *ovata -* the number of cells (*Ostreopsis* sp. 1: *O.* cf. *ovata*) isolated between 22.0°C and 26.9°C were 70∶14, while between 27.0°C and 34.9°C were 46∶58. If this tendency truly reflects their physiological trait, the lack of information on *Ostreopsis* sp. 1 from previous reports could be explained by the fact that all the previous intensive surveys on the genus focused on warmer waters. Thus, extended sampling toward higher latitude may uncover a wider distribution of *Ostreopsis* sp. 1.

The evolutionary divergence of the ITS within *O.* cf. *ovata* is more than four times greater than that within *Ostreopsis* sp. 1 (21.94 vs 5.00 in Table 2), although there is no significant difference in the less variable D8/D10. If the evolutionary divergence of the ITS more or less reflects the genomic heterogeneity in each clade, and considering the idea that the genetic diversity in a population reflects its potential to adapt to changing environments [36], [37], it is likely that *O.* cf. *ovata* has greater metabolic potentialities and ecological versatility relative to the genetically more homogeneous *Ostreopsis* sp. 1. This would enhance the possibility to survive successfully under changes of various environmental parameters. Comparative culture experiments with *O.* cf. *ovata* and *Ostreopsis* sp. 1 are currently ongoing (Yamaguchi et al., submitted).

It is not surprising if *Ostreopsis* sp. 1 has been observed before in Japan or elsewhere, but simply identified as *O. ovata* due to its cryptic morphology. The extent to which diversity of *O. ovata* species-complex has previously been neglected/overlooked might be becoming apparent from the increased use of molecular techniques, as it has happened for many other microorganisms (e.g. [38], [39], [40], [41], [42], see also [43]).

### Taxonomic implications

Our phylogenetic trees inferred from D8–D10 and ITS sequence analyses clearly demonstrated that *Ostreopsis* consisted of 8 distinct clades, whose monophylies were recovered in both phylogenies with strong statistic supports. In this paper we provisionally named these clades as a rank of species. Some robust subclades are nested in the clades, such as D-1 and D-2 in *Ostreopsis* sp. 6 in the D8–D10 tree. Nonetheless, we left such clades undivided since finer division may create many paraphyletic groups and each clone must then bear its own name which would be confusing and uninformative except for the clones with prominent divergence, viz. CAWD179, CAWD184 and OdoOst6. Thus, the names refer to the minimum monophyletic units that cannot further be subdivided unless introducing new names. Although we do not imply that all the clades should be accorded to the rank of species in a formal classification, we believe that some (e.g. *Ostreopsis* sp. 1 and *O.* cf. *siamensis*) likely represent the rank of species considering their low level of intra clade divergence in the D8–D10 and the ITS phylogenies.

Based on the observations under LM and SEM, three clades, *O.* cf. *ovata*, *Ostreopsis* sp. 1 and a clone OdoOst6, fitted into the morphological definition of *O. ovata*, which is therefore a species complex involving at least three cryptic species. Cryptic nature of the *O. ovata* species-complex may indicate optimal phenotypes subject to strong stabilizing selection [44]. This implies that the particular forms of this species-complex are functionally relevant to their survival. Although in this study we detected no morphological differences among three clades, we still assume that there are quantitative differentiations at fine structural level so that morphometric analysis or geometric morphometry for thecal plates may uncover subtle differences that are capable of discriminating them. In fact, there is no truly cryptic species had been reported in diatoms, only species that were very difficult to tell apart by eye [34]. Some species initially distinguished on the basis of molecular or mating data will often subsequently be found to exhibit small morphological differences as reported so far in dinoflagellate [45] as well as coccolithophorid [46], diatoms [47], [48] and foraminifer [49].

Even if more detailed morphological studies, molecular sequences and/or mating experiments delineate the species border among *O.* cf. *ovata*, *Ostreopsis* sp. 1 and the clone OdoOst6, the formal species description should be postponed until we make certain of which clade bears the name *O. ovata*. Based on the materials collected from French Polynesia, New Caledonia and the Ryukyu Islands, *O. ovata* was described by Fukuyo [50] in that the species description lacked the designation of holotype and type locality. Thus, it is necessary to examine the materials from one (or preferably all) of sites where Fukuyo collected and described *O. ovata* for the 1981 paper [50] in order to clarify if 1) clones collected from these sites are genetically homogenous, i.e. the assemblage solely comprises *O.* cf. *ovata*, *Ostreopsis* sp. 1 or *Ostreopsis* sp. 2 clade, 2) they are homogenous within *O. ovata* species-complex but form its own clade, or 3) they are heterogeneous (i.e. the assemblage comprises more than one clade). The scenarios 1) and 2) are straightforward: one clade, to which materials from the original localities belong, will bear the name *O. ovata* and the others need new name. On the other hand, if the scenario 3) is the case, and this is quite plausible judging from the result that ca. 25% of our sample are heterogeneous (17 out of 65 samples), one clade will arbitrary be selected as *O. ovata* with one representative clone designated as a neotype (or lectotype if possible).

There is also distinct phylogenetic structure within the clade of *Ostreopsis* sp. 6, indicated D-1 and D-2 in the D8–D10 tree. The uncorrected genetic distance (*p*) obtained from the ITS sequence between them was 0.222. Litaker et al. [51] indicated with ITS variation among 14 genera of dinoflagellate that *p* values between 0.042 and 0.580 substitutions per site in this region are indicative of species level divergence. Recently Litaker et al. [52] confirmed the idea with *Gambierdiscus*, where two morphologically similar but barely distinguishable species *G. yasumotoi* and *G. ruetzleri* had p>0.07. A newly described species *Coolia malayensis*, bearing distinct morphology, was separated from *C. monotis* with p = 0.117, whereas intra-specific values were <0.004 and 0.028, respectively [53]. Incidentally, although two ITS sequences AF218465 and FM244728 are annotated as *O. lenticularis* and *O.* cf. *labens*, respectively, these sequences were closely related in our ITS phylogeny in the same clade and the branch length separating them are extremely short (see Fig. 2). The *p* value between AF218465 and FM244728 was 0.0079 (3 sites in 377 bp), which is largely below species level divergence according to Litaker et al. [51]. The conspecificity of these sequences is also supported by the absence of CBCs in their ITS2, in which the presence CBC has been proposed as an indication of species level divergence in eukaryota [54].

Together with OdoOst6 in *O. ovata* species-complex, 2 clones collected from Oceania area (CAWD179 from Australia, CAWD184 from Cook Islands) are also highly diverged; presumably each of them corresponds to a rank of species. Taxonomic study is now ongoing for these clones (Smith and Rhodes, unpubl.).

### Toxicity

This study added new information regarding *Ostreopsis* distribution, and thus where palytoxin-like poisoning can potentially take place. Agreeing with previous reports of *Ostreopsis* toxicities (e.g. [25]), most of the clones examined in this study exhibited mouse toxicity, reconfirming the urgent need of developing the early detection system for the toxic *Ostreopsis*.

The mouse toxicity of a clone s0716, belonging to the most abundantly occurring species, *Ostreopsis* sp. 1, was the highest among the clones tested in this assay. The clone s0716 was collected from Otsuki Town, Kochi (JT1 in Fig. 5), from where serranidaen fish were caught and consumed and caused PTX-like symptom for 11 out of 33 people in 2000 [55]. In 2007, 2 out of 4 people caused PTX-like symptom after eating ostraciidaen fish collected from Fukue Island, Nagasaki [12], from where we collected the sample JS1 and all the 12 clones isolated were exclusively *Ostreopsis* sp. 1. The PTX-like symptoms, however, should not necessarily be linked to the occurrence of the highly toxic *Ostreopsis* sp. 1 unless direct evidences is available since our study demonstrated that a distance of only several meters can alter the clade composition as shown JP1-3; JR1-3; JQ1-3 in Fig. 5. Nevertheless, we believe that it is worthwhile paying particular attention to those areas with high *Ostreopsis* sp. 1 records to monitor the occurrence of *Ostreopsis* sp. 1 and minimize the potential risk of the poisoning.

Recently PTX-like compound (Ovatoxin-a like new compound) was detected using LC-MS/MS from our clones belonging to *O.* cf. *ovata* and *Ostreopsis* sp.1 and Ostreocin-D was detected from the clone belonging to *Ostreopsis* sp.6 (s0587), whereas no PTX-like compound was detected from the clones belonging to *Ostreopsis* sp.5 (Suzuki et al. submitted), which support results of toxicities of our clones by mouse bioassay.

### Comparison of genetic markers

High copy number, together with often large intragenomic polymorphism levels, make orthology virtually impossible to determine at the outset, the result being that ITS has the strong potential to obscure species boundaries [56] and biodiversity estimates [57].

Highly divergent ITS sequences of *Ostreopsis* made reliable alignment intricate. Difficulty of ribosomal DNA alignment can sometimes be overcome when the homology assessment is feasible referring to the secondary structure model of its transcribed RNA [58]; however, our preliminary secondary structure prediction (not shown) using RNAstructure 4.5 [59] failed to recover the common structural features ([54]; see also [60]). Therefore, we decided not to use the secondary structure for the aid of the ITS alignment.

The discrepancies of the ITS topologies using the different alignment algorisms poses the question as to whether the ITS region is appropriate for screening the phylogeography of *Ostreopsis*. The frequent need to clone sequences is indicative of the presence of polymorphisms in the ITS copies obtained from *Ostreopsis* isolates, and is disadvantageous in terms of time and cost when large number of samples require screening.

Regarding the phylogenetic information, both markers used in this study have sufficient resolution to separate the clades which likely corresponds to a rank of species in *Ostreopsis*. The ITS, however, is too variable at least for *Ostreopsis* to securely reconstruct the relationship among the clades, whereas the less variable D8–D10 allows resolution of the inter clade topology. On the other hand, the ITS is suitable to look at shallow level (recent) diversification, i.e. comparison of local populations within *O.* cf. *ovata* clade, that cannot be determined with the D8–D10 which provides little information about the internal divergence of each clade.

The D1–D2 is also a gene maker that has widely been used for different level of comparisons in wide range of organisms. This region is known to be less suitable to resolve species level comparison. For example, in a gonyaulacalean dinoflagellate, *Gambierdiscus* [52], the genus first separated into clades but only fully resolved in the D8–D10 tree, with subsequent divergence order into 8 species (*G. pacificus*, *G. toxicus*, *G. belizeanus*, *G. carpenteri*, *G. caribaeus*, *G. australes*, *G. carolinianus* and *G. polynesiensis*). The result obtained by Penna et al. [25] showed that the variability of the D1–D2 of *Ostreopsis* is intermediate between ITS and D8–D10. The internal topology of the *O.* cf. *ovata* clade was resolved in their ML tree based on D1–D2 sequences.

As a whole, LSU marker, D1–D2 or D8–D10, is recommended for further use. The D1–D2 may suit for finer level comparison such as phylogeographic survey, whereas D8–D10 would be appropriate for species-level comparison such as molecular systematics/taxonomy within the genus. Both can be used for the practical use, i.e. molecular-based monitoring via fluorescence *in situ* hybridization, microarray or quantitative real-time PCR.

## Materials and Methods

### Ethics Statement

No specific permits were required for the sampling as the locations are not privately-owned or protected in any way, and the field studies did not involve endangered or protected species. The Animal Use Protocol (AUP) for handling mouse described here was approved by the Animal Ethics Committee (approval ID: D-00068) of Kochi University.

### Sampling and culture

Algal substrata were collected mainly from middle to southern part of Japan as well as from the other geographic regions. The details of samples, including locality and water temperature, if available, are shown in Table S1. In the laboratory the seaweeds were vigorously shaken to cause epiphytes, including *Ostreopsis*, to detach from the substrata. The resultant suspension was sieved twice, firstly through 150 µm and then through 20 µm Nitex mesh. Materials retained on the second mesh were resuspended in filtered seawater and examined under an inverted microscope for cell isolation. Clonal cultures of *Ostreopsis* were established with Provasoli enriched seawater (PES) [61], F/2 [62] or Daigo IMK (Nihon Pharmaceutical Co., Ltd. Tokyo, Japan) media. We tried to isolate as many of the cells of *Ostreopsis* as possible in each sample; therefore the proportion of each clade in a sample should have reflected ± original proportion at a field. There was no difficulty in establishing and/or maintaining cultures of particular clades, so it is unlikely that the culture step introduced a bias for the data analysis. One *Ostreopsis* clone, NIES1404, was purchased from a microbial culture collection at national institute for environmental studies (NIES). All the clones were maintained at 25°C, with 100 µmol photons·m^−2^·s^−1^ from cool-white tubes; the photoperiod was 12∶12 h L∶D. The New Zealand and Australian isolates are maintained in the Cawthron Institute Culture Collection of Micro-algae (CICCM).

### DNA extraction, PCR and sequencing

For the PCR and cloning of ITS, genomic DNA was extracted from cultures in logarithmic growth phase using the DNeasy Plant Kit (Qiagen, Valencia, CA, USA). The 5.8S rDNA and ITS regions (ITS1 and ITS2) were amplified by using 50 µM of oligonucleotide primers ITSA (5′ - GTA ACA AGG THT CCG TAG GT - 3′) and ITSB (5′ - AKA TGC TTA ART TCA GCR GG - 3′) modified after Adachi et al. [63]. The PCR cycling comprised of an initial 5 min heating step at 94°C, followed by 30 cycles of 94°C for 1 min, 55°C for 2 min, and 72°C for 3 min, and a final extension at 72°C for 10 min. The quantity and length of products were examined by agarose gel electrophoresis against known standards. Excess primers and dNTPs were removed from PCR product using High Pure PCR Cleanup Micro Kit (Roche, Tokyo, Japan). The amplified PCR fragments were cloned in the T-vector pMD20 (Takara Bio, Shiga, Japan).

To amplify the D8–D10 region, direct cell PCR approach was used, where intact cells instead of purified genomic DNA were used as template in order to screen large number of clones quicker and more efficiently. 4–6 cells were microscopically collected from culture wells using Pasteur pipette and transferred into a PCR tube. PCR reactions typically contained a 25-µl mixture: 1 µl of MightyAmp DNA Polymerase (1.25 U/µl, Takara Bio, Shiga, Japan); primers as described by Chinain et al. [64]) (7.5 pmol each); 12.5 µl of 2×MightyAmp buffer (Mg^2+^, dNTP plus) which contains Magnesium chloride (4 mM) and dNTPs (800 µM each). In case cell-PCR failed for some clones we extracted DNA and used as a template for standard PCR as for the ITS described above. The PCR cycling comprised of an initial 6 min heating step at 98°C, followed by 25 cycles of 94°C for 30 sec, 55°C for 1 min, and 72°C for 1 min, and a final extension at 72°C for 10 min.

The Big Dye Terminator v3.1 Cycle Sequencing Kit (Applied Biosystems, Tokyo, Japan) was used for sequencing of the ITS clones and the D8–D10 PCR products. Primers and excess dye-labeled nucleotides were removed using the Performa DTR V3 clean-up system (Edge Biosystems, Gaithersburg, MD). Sequencing products were run on an ABI PRISM 3100-Avant Genetic Analyzer (Applied Biosystems). Forward and reverse reads were edited and aligned using SeqMan (DNASTAR, Madison, WI).

All the information of clones, including source sample and accession numbers are listed in Table S2.

### Alignment

In the D8–D10 and the ITS datasets, the 5′ and 3′ ends were manually aligned to truncate and refine the both ends. Three different alignment algorithms, MAFFT [65], Muscle [66] and ClustalW [67], all implemented in Jalview 2.6.1 [68], [69], were used with default settings.

For all the datasets, clones sharing the identical sequences were pruned as redundancies, leaving one sequence as a representative of a ribotype (Table S2). For the D8–D10, which was sequenced directly, the amounts of ambiguously read bases, presumably due to the multicopy and polymorphic nature of the rDNA, were less than 1%.

### Phylogeny

RAxML-VI-HPC, v7.0.4 [70] was used for ML analyses. We conducted a rapid Bootstrap analysis and search for the best-scoring ML tree in one single run with −f a option for 100 repeats. MrBayes 3.1.2 [71], [72] was used for BI to estimate the posterior probability distribution using Metropolis-Coupled Markov Chain Monte Carlo (MCMCMC) [72]. MCMCMC from a random starting tree were used in this analysis with two independent runs and 1 cold and 3 heated chains with temperature set 0.2. Trees were sampled every 100th generation. To increase the probability of chain convergence, we sampled at least 10,000 trees after the standard deviation values of the two runs dipped below 0.01 to calculate the posterior probabilities. Numbers of generations and burn-in are given in Table 1.

MrModeltest 2 [73] was used to determine the most appropriate model of sequence evolution. Selected models and parameters are summarized in Table 1. Selected model was used for ML and BI analyses, but gamma correction values and a proportion of invariable site were obtained by each program, respectively.

### Sequence analyses

To compare the sequence heterogeneity of the D8–D10 and the ITS, CG content was calculated from each dataset.

The number of base differences per sequence from averaging over all sequence pairs between and within each clade was calculated in MEGA4 [74]. All results are based on the pairwise analysis. Standard error estimates were obtained by 1,000 bootstrap replicates. All positions containing alignment gaps and missing data were eliminated only in pairwise sequence comparisons (Pairwise deletion option).

### Mouse bioassay

Crude extracts from methanolic fractions of 6 clones were dried and then dissolved in 1,500 ml of 0.85% saline solution containing 1% Tween 60. Three mice (ddY 20 g) per *Ostreopsis* clone were intraperitoneally administered a single dose extracted from the order of 10^5^ cells in 500 ml. Mice were observed over 48 h and signs and time of death were recorded. Fractions were considered non-toxic if injection of a dose was not lethal to less than 2 mice [7]. If the fraction was toxic to mice, a ten times dilution, viz. 10^4^ cells' extract, was further tested.

### Observation

Living cells under exponential growth phase were observed and measured using Zeiss Axiophoto2 (Zeiss, Oberkochen, Germany) microscopes with bright field optics. Thecal morphology and plate tabulation were examined with an epifluorescence microscope following the calcofluor staining [75]: harvested cells were stained with 1% fluorescent brightner 28 solution (Sigma Chemical, St. Louis, MO) for 30 minutes. For scanning electron microscopy (SEM), cells were fixed with 10% glutaraldehyde and 2% OsO_4_ for 1 h at room temperature, rinsed with distilled water several times, and dehydrated with a ethanol series (70, 90, 99 and 100%×2) followed by t-butyl alcohol series (50, 70, 90, 99 and 100%×2). Cells were frozen in liquid nitrogen and freeze-dried using FRD-82M FREEZE DRYER (IWAKI, Tokyo, Japan). Dried specimens were mounted onto SEM stubs with carbon tape and coated with Pt using a JFC - 1600 AUTO FINE COATER (JEOL, Tokyo, Japan). SEM JSM-6500F (JEOL, Tokyo, Japan) were used at accelerating voltages of 5 kV, and 5 mm working distance.

## Supporting Information

Figure S1 Molecular phylogenies of *Ostreopsis* inferred from D8–D10 sequence. Trees reconstructed from datasets aligned with 3 different algorithms, MAFFT, Muscle and ClustalW, and with two optimally criterion, ML and BI. Trees are rooted with *Coolia* as outgroup. Labels are pruned from major clades. Each color corresponds to phylogenetic trees in Figs. 1 and 2, i.e. (red: *O.* cf. *ovata*, blue: *Ostreopsis* sp. 1, brown: *Ostreopsis* sp. 2, black: *O.* cf. *siamensis*, orange: *Ostreopsis* sp. 4, green: *Ostreopsis* sp. 5, purple: *Ostreopsis* sp. 6).
(TIF)

Figure S2 Molecular phylogenies of *Ostreopsis* inferred from ITS sequence. See caption in Figure S1 for more detail. In ML-MAFFT, probable position of the root, considering the topologies of D8–D10 trees, is indicated by arrow that is used as a pseudo-root point in Fig. 2 to make it superficially similar to the D8–D10 rooted tree and to facilitate the direct comparison between them.
(TIF)

Table S1 Details of samples collected in this study.
(DOC)

Table S2 Details of clones of *Ostreopsis* spp. Sequences obtained in this study are indicated in bold.
(DOC)
