# Supplementary figures and images for: Phylogeography of Ostreopsis along West Pacific Coast, with Special Reference to a Novel Clade from Japan
Source: PLoS One. 2011 Dec 2;6(12):e27983. doi: 10.1371/journal.pone.0027983 (PMC3229513; doi:10.1371/journal.pone.0027983)

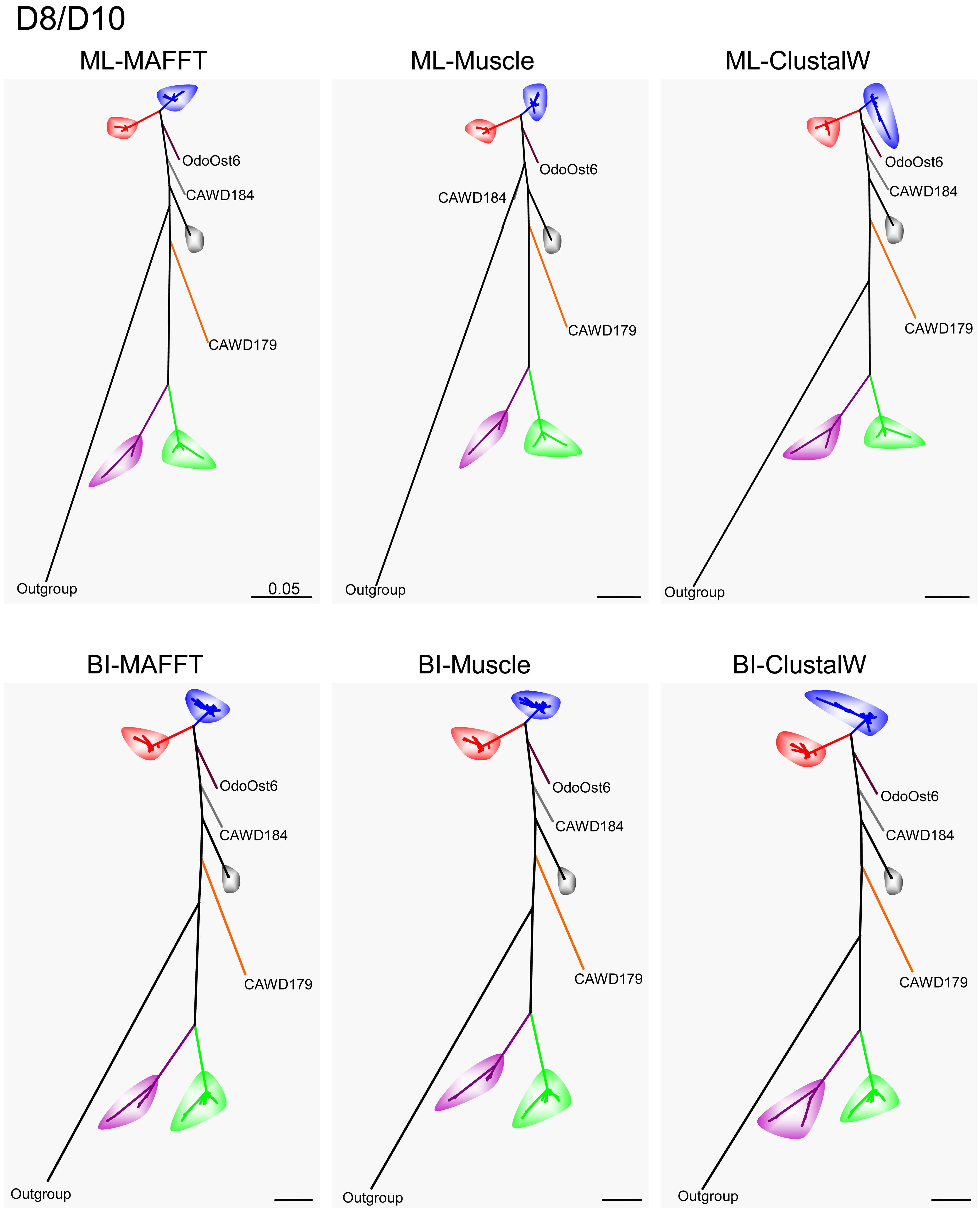

Supplement: Figure S1 — Molecular phylogenies of Ostreopsis inferred from D8–D10 sequence. Trees reconstructed from datasets aligned with 3 different algorithms, MAFFT, Muscle and ClustalW, and with two optimally criterion, ML and BI. Trees are rooted with Coolia as outgroup. Labels are pruned from major clades. Each color corresponds to phylogenetic trees in Figs. 1 and 2, i.e. (red: O. cf. ovata, blue: Ostreopsis sp. 1, brown: Ostreopsis sp. 2, black: O. cf. siamensis, orange: Ostreopsis sp. 4, green: Ostreopsis sp. 5, purple: Ostreopsis sp. 6). (TIF) [file pone.0027983.s001.tif]

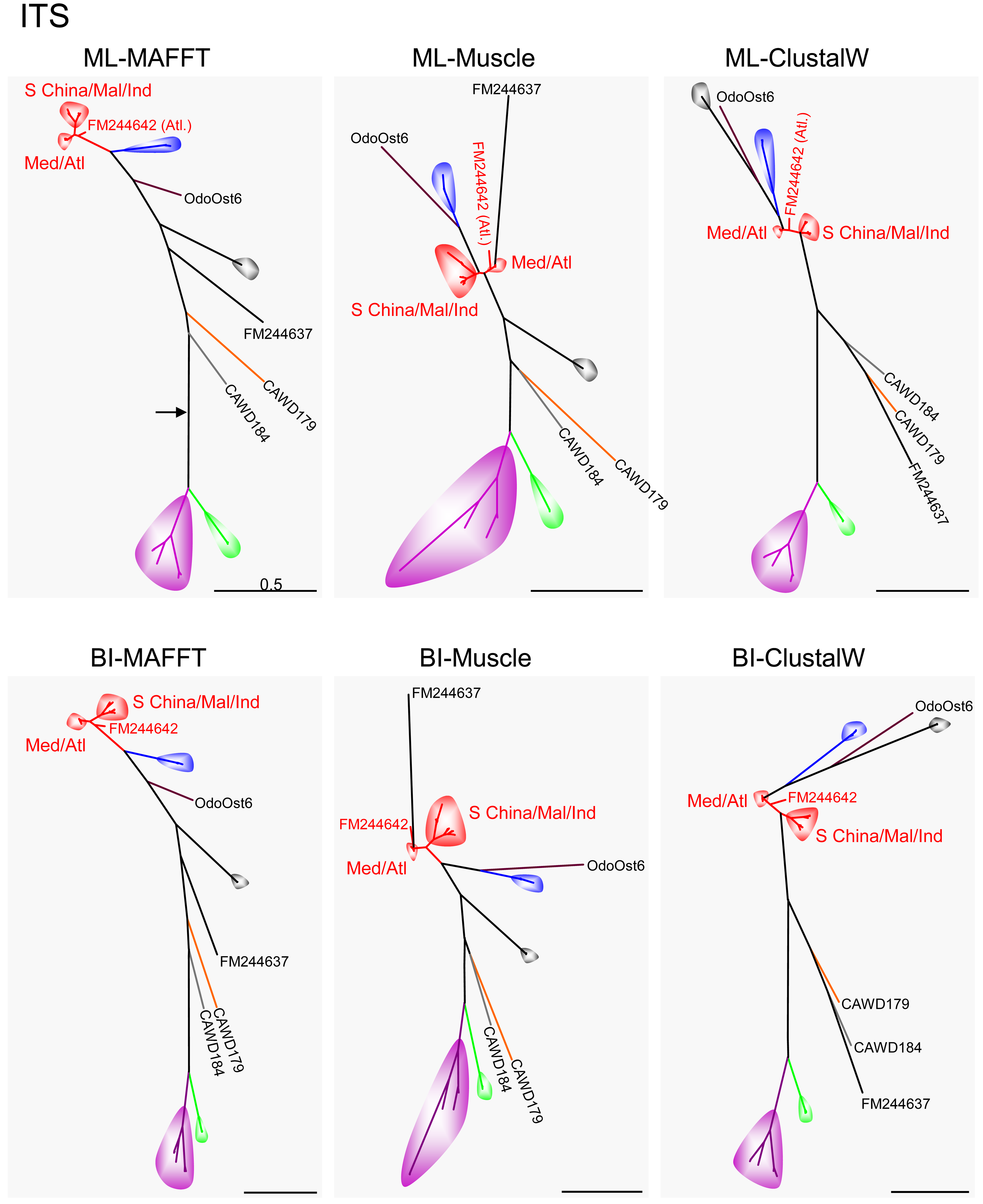

Supplement: Figure S2 — Molecular phylogenies of Ostreopsis inferred from ITS sequence. See caption in Figure S1 for more detail. In ML-MAFFT, probable position of the root, considering the topologies of D8–D10 trees, is indicated by arrow that is used as a pseudo-root point in Fig. 2 to make it superficially similar to the D8–D10 rooted tree and to facilitate the direct comparison between them. (TIF) [file pone.0027983.s002.tif]
