# Supplementary material for: Phylogeography of Ostreopsis along West Pacific Coast, with Special Reference to a Novel Clade from Japan
Source: PLoS One. 2011 Dec 2;6(12):e27983. doi: 10.1371/journal.pone.0027983 (PMC3229513; doi:10.1371/journal.pone.0027983)
Supplement: Table S1 — Details of samples collected in this study. (DOC) [file pone.0027983.s003.doc]

**Table S1.** Details of samples collected in this study.

| Sample | Locality | Collection date | Temp (ºC) | Substratum | Collector |
| --- | --- | --- | --- | --- | --- |
| JK2 | Kubazaki, Kohama Island, Okinawa, Japan | 2009/06/24 |  | *Zostera* | M. Tsuda |
| JK3 | Kubazaki, Kohama Island, Okinawa, Japan | 2009/06/24 |  | *Zostera* | M. Tsuda |
| JK4 | Haemida, Iriomote Island, Okinawa, Japan | 2009/06/24 |  | *Zostera* | M. Tsuda |
| JM2 | Aza-Gima, Yomitan-son, Okinawa, Japan | 2009/06/21 | 29.0 | *Laurencia* | N. Oka |
| JL2 | Uken, Uruma City, Okinawa, Japan | 2009/06/21 | 30.0 | *Liagora mucosissima* | N. Oka |
| JL3 | Uken, Uruma City, Okinawa, Japan | 2010/05/30 | 26.0 | *Garaxaura* | N. Oka |
| JN1 | Nagata cho, Nagasaki city, Nagasaki, Japan | 2009/07/05 | 24.7 | *Gelidium elegans* | T. Fukao |
| JN2 | Kamiura, Nagasaki city, Nagasaki, Japan | 2009/07/05 | 26.4 | *G. elegans* | T. Fukao |
| JN3 | Misaki cho, Nagasaki city, Nagasaki, Japan | 2009/07/05 | 24.4 | *G. elegans* | T. Fukao |
| JN4 | Misaki cho, Nagasaki city, Nagasaki, Japan | 2009/07/05 | 24.4 | *Sargassum* | T. Fukao |
| JO1 | Hidaka, Kagoshima city, Kagoshima, Japan | 2009/07/12 | 27.0 | Sargassum *thumbergii* | T. Yoshikawa |
| MB1 | Bishagoiwa, Muroto city, Kochi, Japan | 2006/10/07 | 25.0 |  | T. Ikegami |
| MB2 | Bishagoiwa, Muroto city, Kochi, Japan | 2006/11/05 | 23.8 |  | T. Ikegami |
| MB3 | Bishagoiwa, Muroto city, Kochi, Japan | 2007/08/31 | 28.7 | *Jania* | T. Ikegami |
| MB4 | Bishagoiwa, Muroto city, Kochi, Japan | 2008/06/14 | 23.7 | *G. elegans* | T. Ikegami |
| MB5 | Bishagoiwa, Muroto city, Kochi, Japan | 2008/08/28 | 27.1 | *G. elegans* | T. Ikegami |
| JP1 | Bishagoiwa, Muroto city, Kochi, Japan (Site A) | 2009/07/11 | 25.1 | *G. elegans* | T. Nishimura |
| JP2 | Bishagoiwa, Muroto city, Kochi, Japan (Site B) | 2009/07/11 | 25.1 | *G. elegans* | S. Sato |
| JP3 | Bishagoiwa, Muroto city, Kochi, Japan (Site C) | 2009/07/11 | 25.1 | *G. elegans* | T. Nishimura |
| JP5 | Bishagoiwa, Muroto city, Kochi, Japan | 2009/08/30 | 28.6 | *G. elegans* | T. Nishimura |
| S1 | Susaki city, Kochi, Japan | 2007/08/30 | 28.0 | *Jania* | T. Ikegami |
| S2 | Susaki city, Kochi, Japan | 2008/06/15 | 23.1 | *G. elegans* | T. Ikegami |
| JQ1 | Susaki city, Kochi, Japan (Site A) | 2009/06/14 | 27.4 | *G. elegans* | T. Nishimura |
| JQ2 | Susaki city, Kochi, Japan (Site B) | 2009/06/14 | 27.4 | *Pterocladiella* | T. Nishimura |
| JQ3 | Susaki city, Kochi, Japan (Site C) | 2009/06/14 | 27.4 | *Tricleocarpa* | T. Nishimura |
| JQ4 | Susaki city, Kochi, Japan | 2009/09/16 | 28.0 | *G. elegans* | T. Nishimura |
| JQ5 | Susaki city, Kochi, Japan | 2009/10/19 | 24.8 | *Vanvoorstia* | S. Sato |
| T1 | Tei, Konan City, Kochi, Japan | 2006/05/29 | 24.4 |  | T. Ikegami |
| T2 | Tei, Konan City, Kochi, Japan | 2006/07/30 | 31.2 |  | T. Ikegami |
| T3 | Tei, Konan City, Kochi, Japan | 2006/08/23 | 31.5 |  | T. Ikegami |
| T4 | Tei, Konan City, Kochi, Japan | 2007/08/28 | 31.8 |  | T. Ikegami |
| T5 | Tei, Konan City, Kochi, Japan | 2008/05/07 | 24.3 | *Collarina pilulifera* | T. Ikegami |
| T6 | Tei, Konan City, Kochi, Japan | 2008/06/24 | 24.9 | *Collarina pilulifera* | T. Ikegami |
| T8 | Tei, Konan City, Kochi, Japan | 2008/09/08 | 30.0 | *G. elegans* | T. Ikegami |
| JR1 | Tei, Konan City, Kochi, Japan (Site A) | 2009/07/28 | 25.9 | *Pterocladiella* | T. Nishimura |
| JR2 | Tei, Konan City, Kochi, Japan (Site B) | 2009/07/28 | 25.9 | *Pterocladiella* | T. Nishimura |
| JR3 | Tei, Konan City, Kochi, Japan (Site C) | 2009/07/28 | 25.9 | *Pterocladiella* | T. Nishimura |
| JR4 | Tei, Konan City, Kochi, Japan | 2009/08/21 | 30.7 | *Pterocladiella* | T. Nishimura |
| JS1 | Tontomari, Fukue Island, Nagasaki, Japan | 2009/07/22 | 26.3 | *Pterocladiella* | T. Nishimura |
| JT1 | Subogata, Otsuki Town, Kochi, Japan | 2009/08/01 | 27.0 | *Tricleocarpa or Pterocladiella or Dictyota* | T. Nishimura |
| JT2 | Issai, Otsuki Town, Kochi, Japan | 2009/08/06 |  |  | T. Okami |
| JT3 | Issai, Otsuki Town, Kochi, Japan | 2009/09/13 | 27.5 | *G. elegans* | T. Okami |
| OH | Higa, Uruma City, Okinawa, Japan | 2010/09/12 | 34.0 | *Marginisporum crassissima* | N. Oka |
| CT | Okinoshima, Tateyama City, Chiba, Japan | 2010/09/07 | 29.2 | *Sargassum fulvellum* | Y. Iwamoto |
| CK | Yoshio, Katsuura City, Chiba, Japan | 2010/09/10 | 22.1 |  | Y. Iwamoto |
| KM | Shirasugi, Maizuru City, Kyoto, Japan | 2010/09/04 | 30.8 |  | H. Nishimura |
| WK | Takatomi, Kushimoto Town, Wakayama, Japan | 2010/05/12 | 24.5 | *Pterocladia capillacea* | S. Komatsu |
| HF1 | Higashi Otaru, Otaru City, Hokkaido, Japan | 2010/10/01 | 20.8 | *Tichocarpus crinitus* | Y. Nomiya |
| HF2 | Higashi Otaru, Otaru City, Hokkaido, Japan | 2010/10/01 | 20.8 | *Fucus* | Y. Nomiya |
| IR | Uehara, Taketomi Town, Iriomote Island, Okinawa, Japan | 2010/04/19 | 24.8 | *Actinotorichia fragilis* | K. Tose |
| Ok1 | Mashiki, Ginowan, Okinawa, Japan | 2010/01/28 |  |  | A. Nakashima |
| Ok2 | Kabira, Ishigaki, Okinawa, Japan | 2010/07/25 |  | *Lithophyllum pygmaeum* | S. Suda |
| Ok3 | Ikeijima, Uruma, Okinawa, Japan | 2008/07/08 |  | *Tricleocarpa cylindrica* | S. Suda |
| Ok4 | Yonashiroyahei, Uruma, Okinawa, Japan | 2010/06/28 |  |  | S. Suda |
| Ok5 | Shiraho, Ishigaki, Okinawa, Japan | 2009/02/27 |  | *Lithophyllum bamleri* | A. Kato |
| Ok6 | Odo, Itoman, Okinawa, Japan | 2009/01/12 |  |  | S. Suda |
| Ok7 | Asato, Nishihara, Okinawa, Japan | 2010/01/21 |  |  | A. Nakashima |
| Kagawa292 | Nishidomari, Otsuki Town, Kochi, Japan | 2007/04/21 |  |  | S. Yoshimatsu |
| Kaiyo | Kaiyo Cho, Tokushima, Japan | 2009/05/12 |  |  | M. Tsuda |
| Italy | La Spezia beach, Genoa, Ligurian Sea, Italy | 2005/08 |  |  | R. Scenati |
| NZ1 | Kerikeri, New Zealand | 1999/05/01 |  |  | North Health Services |
| NZ2 | Mahinepua, New Zealand | 2006/04/01 |  |  | R. Munn |
| NZ3 | Rangaunu Harbour, New Zealand | 2009/02/01 |  |  | S. Waitai & L. Rhodes |
| Australia | Franklin Harbour, South Austlaria | 2009/12 |  |  | C. Wilkinson |
| Cook1 | Rarotonga, Cook Islands | 2009/06/01 |  |  | R. Strickland & A. Sellwood |
| Cook2 | Cook Islands | 2010/07 |  | *Halimeda* sp. | M. Packer |
| NIES | Yaene Harbor Hachijojima Tokyo Japan | 2003/06/27 |  |  | A. Kai |
| Malaysia | Pulau Tinggi, Malaysia | 2008/07 |  |  | G. Usup |
