# Supplementary material for: Phylogeography of Ostreopsis along West Pacific Coast, with Special Reference to a Novel Clade from Japan
Source: PLoS One. 2011 Dec 2;6(12):e27983. doi: 10.1371/journal.pone.0027983 (PMC3229513; doi:10.1371/journal.pone.0027983)
Supplement: Table S2 — Details of clones of Ostreopsis spp. Sequences obtained in this study are indicated in bold. (DOC) [file pone.0027983.s004.doc]

**Table S2.** Details of clones of *Ostreopsis* spp.. Sequences obtained in this study are indicated in bold.

|  |  | D8-D10 |  | ITS |  |
| --- | --- | --- | --- | --- | --- |
| Clone (subclade) | Sample | Accession | Ribotype* | Accession | Ribotype* |
| *Ostreopsis* cf. *ovata* (clade A) |  |  |  |  |  |
| IshiOst61 | Ok5 | **AB674776** | 1 |  |  |
| s0593 | JK2 | as above | 1 |  |  |
| s0623 | JO1 | as above | 1 |  |  |
| s0624 | JO1 | as above | 1 |  |  |
| s0656 | JR1 | as above | 1 |  |  |
| s0669 | JR2 | as above | 1 |  |  |
| s0673 | JR2 | as above | 1 |  |  |
| s0677 | JR3 | as above | 1 |  |  |
| s0678 | JR3 | as above | 1 |  |  |
| s0680 | JR3 | as above | 1 |  |  |
| s0697 | JT1 | as above | 1 |  |  |
| s0699 | JT1 | as above | 1 |  |  |
| s0700 | JT1 | as above | 1 |  |  |
| s0701 | JT1 | as above | 1 |  |  |
| s0715 | JT1 | as above | 1 |  |  |
| s0727 | JT1 | as above | 1 |  |  |
| s0752 | JT2 | as above | 1 |  |  |
| s0756 | JR4 | as above | 1 |  |  |
| s0767 | JT3 | as above | 1 |  |  |
| s0768 | JT3 | as above | 1 |  |  |
| T60730-1 | T2 | as above | 1 | **AB674902** | 22 |
| T60826-2 | T3 | as above | 1 | as above | 22 |
| T70828-2 | T4 | as above | 1 | **AB674903** |  |
| T80908-2 | T8 | as above | 1 |  |  |
| KDo5 (A-1) | Ok4 | **AB674777** | 2 |  |  |
| OdoOst5 (A-1) | Ok6 | as above | 2 |  |  |
| ParkOst2 (A-1) | Ok7 | as above | 2 |  |  |
| s0612 (A-1) | JN1 | as above | 2 |  |  |
| s0619 (A-1) | JN3 | as above | 2 |  |  |
| TroOst4 (A-1) | Ok1 | as above | 2 |  |  |
| YoshiOst3 (A-1) | Ok7 | as above | 2 |  |  |
| s0651 (A-1) | JM2 | **AB674778** | 3 |  |  |
| s0652 (A-1) | JM2 | as above | 3 |  |  |
| s0682 | JR3 | **AB674779** | 4 |  |  |
| s0714 | JT1 | as above | 4 |  |  |
| s0718 | JT1 | as above | 4 |  |  |
| s0710 | JT1 | **AB674780** | 5 |  |  |
| s0731 | JT1 | as above | 5 |  |  |
| s0625 | JO1 | **AB674781** | 6 |  |  |
| s0667 | JR2 | as above | 6 |  |  |
| s0579 (A-2) | JK2 | **AB674782** | 7 | as CAWD174 | 26 |
| s0603 (A-2) | JN1 | as above | 7 |  |  |
| s0698 | JT1 | **AB674783** | 8 |  |  |
| s0706 | JT1 | as above | 8 |  |  |
| s0707 | JT1 | as above | 8 |  |  |
| s0712 | JT1 | as above | 8 |  |  |
| s0723 | JT1 | as above | 8 |  |  |
| s0724 | JT1 | as above | 8 |  |  |
| s0743 | JT2 | as above | 8 |  |  |
| s0703 | JT1 | **AB674784** | 9 |  |  |
| s0722 | JT1 | as above | 9 |  |  |
| s0726 | JT1 | as above | 9 |  |  |
| s0730 | JT1 | as above | 9 |  |  |
| CAWD174 (A-2) | Cook1 | **AB674785** |  | **AB674904** | 26 |
| OVPT2 (A-2) | Malaysia | **AB674786** |  | **AB674905** |  |
| kab013 | Ok2 | **AB674787** |  |  |  |
| KAC85 | Italy | **AB674788** |  | **AB674906** |  |
| OH23 (A-1) | OH | **AB674789** |  |  |  |
| s0580 (A-2) | JK2 | **AB674790** |  |  |  |
| s0626 | JO1 | **AB674791** |  |  |  |
| s0662 | JR1 | **AB674792** |  |  |  |
| s0679 | JR3 | **AB674793** |  |  |  |
| s0683 | JR3 | **AB674794** |  |  |  |
| s0704 | JT1 | **AB674795** |  |  |  |
| s0705 | JT1 | **AB674796** |  |  |  |
| s0709 | JT1 | **AB674797** |  |  |  |
| s0711 | JT1 | **AB674798** |  |  |  |
| s0713 | JT1 | **AB674799** |  |  |  |
| s0732 | JT1 | **AB674800** |  |  |  |
| s0758 | JR4 | **AB674801** |  |  |  |
| s0759 | JR4 | **AB674802** |  |  |  |
| s0760 | JR4 | **AB674803** |  |  |  |
| s0761 | JR4 | **AB674804** |  |  |  |
| s0763 | JT3 | **AB674805** |  |  |  |
| s0764 | JT3 | **AB674806** |  |  |  |
| s0765 | JT3 | **AB674807** |  |  |  |
| s0766 | JT3 | **AB674808** |  |  |  |
| s0769 | JT3 | **AB674809** |  |  |  |
| s0770 | JT3 | **AB674810** |  |  |  |
| s0771 | JT3 | **AB674811** |  |  |  |
| s0788 | JQ4 | **AB674812** |  |  |  |
| TroOstC (A-1) | Ok1 | **AB674813** |  |  |  |
| TroOstF (A-1) | Ok1 | **AB674814** |  |  |  |
| S70830-4 | S1 | **AB674907** |  |  |  |
| ARPAGenova | [25] |  |  | FM244628 | 21 |
| ARPAL-1 | [25] |  |  | FM994901 | 21 |
| CBA-A | [25] |  |  | FM244621 | 21 |
| CBA-D | [25] |  |  | FM244623 | 21 |
| CBA-E | [25] |  |  | FM244624 | 21 |
| CBA-F | [25] |  |  | FM244625 | 21 |
| CBA-G | [25] |  |  | FM244626 | 21 |
| CBA-P | [25] |  |  | FM244632 | 21 |
| CBA-T | [25] |  |  | FM244633 | 21 |
| CNR-A1 | [20] |  |  | AJ311520 | 21 |
| CNR-D1 | [20] |  |  | AJ320179 | 21 |
| CNR-Z1 | [20] |  |  | AJ420005 | 21 |
| Geo2 | [25] |  |  | FM244627 | 21 |
| IFR-OST01M | *** |  |  | FJ905897 | 21 |
| IFR-OST01V | *** |  |  | FJ905896 | 21 |
| KC34 | [25] |  |  | FM242104 | 21 |
| KC36 | [25] |  |  | FM242105 | 21 |
| KC38 | [25] |  |  | FM242106 | 21 |
| KC39 | [25] |  |  | FM242107 | 21 |
| KC68 | [25] |  |  | FM244732 | 21 |
| KC69 | [25] |  |  | FM244733 | 21 |
| KC70 | [25] |  |  | FM244734 | 21 |
| KC71 | [25] |  |  | FM244735 | 21 |
| OS01BR | [25] |  |  | AJ420006 | 21 |
| OS02BR | [25] |  |  | AJ318461 | 21 |
| OS03BR | [25] |  |  | AJ491311 | 21 |
| OS05BR | [25] |  |  | FM244665 | 21 |
| OS06BR | [20] |  |  | AJ491312 | 21 |
| OS07BR | [25] |  |  | FM244666 | 21 |
| OS10BR | [25] |  |  | FM244667 | 21 |
| OS13BR | [25] |  |  | FM244668 | 21 |
| OS15BR | [20] |  |  | AJ318462 | 21 |
| OS18BR | [25] |  |  | FM244670 | 21 |
| OS20BR | [25] |  |  | FM244671 | 21 |
| Porto Romano | [25] |  |  | FM244630 | 21 |
| VGO822 | [25] |  |  | FM244635 | 21 |
| VGO884 | [25] |  |  | FM244639 | 21 |
| VGO886 | [25] |  |  | FM244640 | 21 |
| VGO887 | [25] |  |  | FM244641 | 21 |
| PD06 | [24] |  |  | AF218455 | 24 |
| PD07 | [24] |  |  | AF218456 | 24 |
| CBA-4 | [25] |  |  | FM244724 | 23 |
| CBA-6 | [25] |  |  | FM244725 | 23 |
| CBA-9 | [25] |  |  | FM244726 | 23 |
| CBA-10 | [25] |  |  | FM244727 | 23 |
| PR01 | [24] |  |  | AF218457 | 25 |
| PR02 | [24] |  |  | AF218459 | 25 |
| PR04 | [24] |  |  | AF218458 | 25 |
| SA02 | [24] |  |  | AF218460 | 27 |
| SA04 | [24] |  |  | AF218461 | 27 |
| ARPA-Lazio | [25] |  |  | FM244629 |  |
| CBA-C | [25] |  |  | FM244622 |  |
| CBA-N | [25] |  |  | FM244631 |  |
| OS04BR | [25] |  |  | FM244664 |  |
| OS16BR | [25] |  |  | FM244669 |  |
| PD04 | [24] |  |  | AF076217 |  |
| PR03 | [24] |  |  | AF076218 |  |
| SA06 | [24] |  |  | AF218463 |  |
| SA09 | [24] |  |  | AF218462 |  |
| SA10 | [24] |  |  | AF218464 |  |
| VGO614 | [25] |  |  | FM244642 |  |
| VGO693 | [25] |  |  | FM244636 |  |
| VGO820 | [25] |  |  | FM244634 |  |
| VGO881 | [25] |  |  | FM244637 |  |
| VGO883 | [25] |  |  | FM244638 |  |
| *Ostreopsis* sp. 1 (clade B) |  |  |  |  |  |
| s0659 (B-1) | JR1 | **AB674815** | 10 |  |  |
| s0666 (B-1) | JR2 | as above | 10 |  |  |
| T80507-5 (B-1) | T5 | **AB674816** | 11 |  |  |
| s0638 (B-1) | JQ2 | as above | 11 |  |  |
| s0642 (B-1) | JQ3 | as above | 11 |  |  |
| s0675 (B-1) | JR3 | as above | 11 |  |  |
| s0676 (B-1) | JR3 | as above | 11 |  |  |
| s0629 (B-1) | JP1 | **AB674817** | 12 |  |  |
| s0663 (B-1) | JR1 | as above | 12 |  |  |
| s0738 (B-1) | JT2 | as above | 12 |  |  |
| s0607 (B-1) | JN1 | **AB674818** | 13 |  |  |
| s0716 (B-1) | JT1 | as above | 13 |  |  |
| s0720 (B-1) | JT1 | as above | 13 |  |  |
| s0721 (B-1) | JT1 | as above | 13 |  |  |
| T70828-5 | T4 | **AB674819** | 14 | **AB674908** |  |
| MB80828-3 | MB5 | as above | 14 | as MB61105 | 28 |
| MB61007-3 | MB1 | as above | 14 | **AB674909** |  |
| MB70831-3 | MB3 | as above | 14 |  |  |
| MB80614-3 | MB4 | as above | 14 |  |  |
| MB80614-4 | MB4 | as above | 14 |  |  |
| MB80614-5 | MB4 | as above | 14 |  |  |
| MB80828-1 | MB5 | as above | 14 |  |  |
| NIES1404^**^ | Hachijojima | as above | 14 |  |  |
| s0640 | JQ2 | as above | 14 |  |  |
| s0644 | JQ3 | as above | 14 |  |  |
| s0668 | JR2 | as above | 14 |  |  |
| s0681 | JR3 | as above | 14 |  |  |
| s0693 | JS1 | as above | 14 |  |  |
| s0750 | JT2 | as above | 14 |  |  |
| s0772 | JP5 | as above | 14 |  |  |
| S70830-3 | S1 | as above | 14 |  |  |
| S80615-2 | S2 | as above | 14 |  |  |
| T80507-3 | T5 | as above | 14 |  |  |
| T80507-4 | T5 | as above | 14 |  |  |
| T80624-6 | T6 | as above | 14 |  |  |
| s0605 | JN1 | **AB674820** | 15 |  |  |
| s0616 | JN3 | as above | 15 |  |  |
| s0620 | JN3 | as above | 15 |  |  |
| s0600 | JN1 | **AB674821** | 16 |  |  |
| s0617 | JN3 | as above | 16 | **AB674910** |  |
| s0609 | JN1 | as above | 16 |  |  |
| s0610 | JN1 | as above | 16 |  |  |
| s0613 | JN1 | as above | 16 |  |  |
| s0728 | JT1 | as above | 16 |  |  |
| s0736 | JT2 | as above | 16 |  |  |
| s0745 | JT2 | as above | 16 |  |  |
| s0746 | JT2 | as above | 16 |  |  |
| s0754 | JT2 | as above | 16 |  |  |
| s0755 | JT2 | as above | 16 |  |  |
| s0696 | JS1 | **AB674822** | 17 |  |  |
| s0725 | JT1 | as above | 17 |  |  |
| s0739 | JT2 | as above | 17 |  |  |
| s0748 | JT2 | as above | 17 |  |  |
| s0751 | JT2 | as above | 17 |  |  |
| MB61105 | MB2 | **AB674823** | 18 | **AB674911** | 28 |
| s0599 | JN1 | as above | 18 |  |  |
| s0708 | JT1 | as above | 18 |  |  |
| s0611 | JN1 | **AB674824** | 19 |  |  |
| s0695 | JS1 | as above | 19 |  |  |
| s0744 | JT2 | as above | 19 |  |  |
| s0747 | JT2 | as above | 19 |  |  |
| HF59 (B-2) | HF1 | **AB674825** |  |  |  |
| HF66 (B-2) | HF2 | **AB674826** |  |  |  |
| kco001 (B-1) | Kaiyo | **AB674827** |  |  |  |
| KM24 (B-2) | KM | **AB674828** |  |  |  |
| MB80614-2 | MB4 | **AB674829** |  |  |  |
| s0598 (B-1) | JN1 | **AB674830** |  |  |  |
| s0601 | JN1 | **AB674831** |  |  |  |
| s0602 | JN1 | **AB674832** |  |  |  |
| s0604 | JN1 | **AB674833** |  |  |  |
| s0606 (B-1) | JN1 | **AB674834** |  |  |  |
| s0608 (B-1) | JN1 | **AB674835** |  |  |  |
| s0615 | JN2 | **AB674836** |  |  |  |
| s0621 (B-1) | JN3 | **AB674837** |  |  |  |
| s0622 (B-1) | JN4 | **AB674838** |  |  |  |
| s0631 (B-1) | JP2 | **AB674839** |  |  |  |
| s0634 (B-1) | JP3 | **AB674840** |  |  |  |
| s0635 (B-1) | JQ1 | **AB674841** |  |  |  |
| s0639 | JQ2 | **AB674842** |  |  |  |
| s0641 (B-1) | JQ2 | **AB674843** |  |  |  |
| s0643 (B-1) | JQ3 | **AB674844** |  |  |  |
| s0645 | JQ3 | **AB674845** |  |  |  |
| s0655 (B-1) | JL2 | **AB674846** |  |  |  |
| s0660 (B-1) | JR1 | **AB674847** |  |  |  |
| s0661 | JR1 | **AB674848** |  |  |  |
| s0670 (B-1) | JR2 | **AB674849** |  |  |  |
| s0671 (B-1) | JR2 | **AB674850** |  |  |  |
| s0684 | JS1 | **AB674851** |  |  |  |
| s0685 | JS1 | **AB674852** |  |  |  |
| s0686 (B-1) | JS1 | **AB674853** |  |  |  |
| s0689 | JS1 | **AB674854** |  |  |  |
| s0690 | JS1 | **AB674855** |  |  |  |
| s0691 (B-1) | JS1 | **AB674856** |  |  |  |
| s0692 | JS1 | **AB674857** |  |  |  |
| s0694 | JS1 | **AB674858** |  |  |  |
| s0717 (B-1) | JT1 | **AB674859** |  |  |  |
| s0733 | JT2 | **AB674860** |  |  |  |
| s0734 (B-1) | JT2 | **AB674861** |  |  |  |
| s0735 (B-1) | JT2 | **AB674862** |  |  |  |
| s0737 (B-1) | JT2 | **AB674863** |  |  |  |
| s0741 (B-1) | JT2 | **AB674864** |  |  |  |
| s0742 (B-1) | JT2 | **AB674865** |  |  |  |
| s0749 (B-1) | JT2 | **AB674866** |  |  |  |
| s0753 (B-1) | JT2 | **AB674867** |  |  |  |
| s0776 | JP5 | **AB674868** |  |  |  |
| s0814 | JQ5 | **AB674869** |  |  |  |
| S80615-1 | S2 | **AB674870** |  |  |  |
| T60529 | T1 |  |  | **AB674912** |  |
| T80624-2 | T6 | **AB674871** |  |  |  |
| CK15 | CK | **AB674872** |  |  |  |
| CT14 (B-2) | CT | **AB674873** |  |  |  |
| CT7 (B-2) | CT | **AB674874** |  |  |  |
| WK23 (B-2) | WK | **AB674875** |  |  |  |
| *Ostreopsis* sp. 2 |  |  |  |  |  |
| OdoOst6 | Ok6 | **AB674876** |  | **AB674913** |  |
| *Ostreopsis* sp. 3 |  |  |  |  |  |
| CAWD184 | Cook2 | **AB674877** |  | **AB674914** |  |
| *O.* cf. *siamensis* |  |  |  |  |  |
| CAWD96 | NZ1 | **AB674878** |  | **AB674915** | 30 |
| CAWD147 | NZ2 | **AB674879** |  | as above | 30 |
| CAWD173 | NZ3 | **AB674880** |  | as above | 30 |
| CNR-B4 | [20] |  |  | AJ301643 | 29 |
| CNR-B5 | [20] |  |  | AJ491335 | 29 |
| CNR-T5 | [25] |  |  | FM244729 | 29 |
| CSIC-D1 | [20] |  |  | AJ491333 | 29 |
| CSIC-D5 | [20] |  |  | AJ312944 | 29 |
| CSIC-D7 | [20] |  |  | AJ491334 | 29 |
| OS1V | [20] |  |  | AJ319871 | 29 |
| OS2V | [20] |  |  | AJ491313 | 29 |
| OS3V | [20] |  |  | AJ491332 | 29 |
| *Ostreopsis* sp. 4 |  |  |  |  |  |
| CAWD179 | Australia | **AB674881** |  | **AB674916** |  |
| *Ostreopsis* sp. 5 (clade C) |  |  |  |  |  |
| MB80828-4 (C-2) | MB5 | **AB674882** |  | **AB674917** |  |
| O70421-1 | Kagawa292 | **AB674883** |  | **AB674918** |  |
| O70421-2 (C-2) | Kagawa292 | **AB674884** |  | **AB674919** |  |
| IkeOst2 (C-1) | Ok3 | **AB674885** |  |  |  |
| MB80828-2 | MB5 | **AB674886** |  |  |  |
| O70421-3 | Kagawa292 | **AB674887** |  |  |  |
| s0577 (C-1) | JK4 | **AB674888** |  |  |  |
| s0578 (C-1) | JK4 | **AB674889** |  |  |  |
| s0627 | JP1 | **AB674890** |  |  |  |
| s0780 (C-3) | JQ4 | **AB674891** |  |  |  |
| s0806 (C-3) | JQ5 | **AB674892** |  |  |  |
| s0808 (C-3) | JQ5 | **AB674893** |  |  |  |
| s0809 (C-3) | JQ5 | **AB674894** |  |  |  |
| *Ostreopsis* sp. 6 (clade D) |  |  |  |  |  |
| IR33 (D-1) | IR | **AB674895** | 20 | **AB674920** |  |
| IR29 (D-1) | IR | as above | 20 |  |  |
| OU11 (D-1) | JL3 | **AB674896** |  | **AB674921** |  |
| s0587 (D-2) | JK4 | **AB674897** |  | **AB674922** |  |
| IR49 | IR | **AB674898** |  |  |  |
| OU8 | JL3 | **AB674899** |  |  |  |
| s0595 (D-2) | JK2 | **AB674900** |  |  |  |
| OLPR01 | [24] |  |  | AF218465 |  |
| VGO897 | [25] |  |  | FM244728 |  |
| *Coolia* sp. |  |  |  |  |  |
| s0584 | JK3 | **AB674901** |  |  |  |

^*^Ribotype name is designated only when multiple clones share the same sequence.

^**^Annotated as *O. siamensis* in NIES microbial culture collection (<http://mcc.nies.go.jp/localeAction.do?lang=en>).

^***^Unpublished sequences directly retrieved from GenBank
